# Novel Genomic Variants, Atypical Phenotypes and Evidence of a Digenic/Oligogenic Contribution to Disorders/Differences of Sex Development in a Large North African Cohort

**DOI:** 10.3389/fgene.2022.900574

**Published:** 2022-08-30

**Authors:** Housna Zidoune, Asmahane Ladjouze, Djalila Chellat-Rezgoune, Asma Boukri, Scheher Aman Dib, Nassim Nouri, Meryem Tebibel, Karima Sifi, Noureddine Abadi, Dalila Satta, Yasmina Benelmadani, Joelle Bignon-Topalovic, Maeva El-Zaiat-Munsch, Anu Bashamboo, Ken McElreavey

**Affiliations:** ^1^ Human Developmental Genetics Unit, Institut Pasteur, CNRS, Paris, France; ^2^ Laboratory of Molecular and Cellular Biology, Department of Animal Biology, University Frères Mentouri Constantine 1, Constantine, Algeria; ^3^ Department of Medicine, Laboratory of Biology and Molecular Genetics, University Salah Boubnider Constantine 3, Constantine, Algeria; ^4^ Department of Pediatrics, CHU Bab El Oued, Algiers, Algeria; ^5^ Department of Endocrinology and Diabetology, CHU Ibn Badis Constantine, Constantine, Algeria; ^6^ Nissia Djebel Ouahch Clinic, Constantine, Algeria; ^7^ Department of Pediatric Surgery, CHU Beni Messous, Algiers, Algeria

**Keywords:** disorders/differences in sex development (DSD), gonadal dysgenesis, genetic etiology, digenic/oligogenic, testis, ovary, genitalia

## Abstract

In a majority of individuals with disorders/differences of sex development (DSD) a genetic etiology is often elusive. However, new genes causing DSD are routinely reported and using the unbiased genomic approaches, such as whole exome sequencing (WES) should result in an increased diagnostic yield. Here, we performed WES on a large cohort of 125 individuals all of Algerian origin, who presented with a wide range of DSD phenotypes. The study excluded individuals with congenital adrenal hypoplasia (CAH) or chromosomal DSD. Parental consanguinity was reported in 36% of individuals. The genetic etiology was established in 49.6% (62/125) individuals of the total cohort, which includes 42.2% (35/83) of 46, XY non-syndromic DSD and 69.2% (27/39) of 46, XY syndromic DSD. No pathogenic variants were identified in the 46, XX DSD cases (0/3). Variants in the *AR, HSD17B3, NR5A1* and *SRD5A2* genes were the most common causes of DSD. Other variants were identified in genes associated with congenital hypogonadotropic hypogonadism (CHH), including the *CHD7* and *PROKR2*. Previously unreported pathogenic/likely pathogenic variants (n = 30) involving 25 different genes were identified in 22.4% of the cohort. Remarkably 11.5% of the 46, XY DSD group carried variants classified as pathogenic/likely pathogenic variant in more than one gene known to cause DSD. The data indicates that variants in *PLXNA3*, a candidate CHH gene, is unlikely to be involved in CHH. The data also suggest that *NR2F2* variants may cause 46, XY DSD.

## Introduction

Disorders/differences of sex development (DSD) are defined as heterogeneous congenital conditions associated with discordant development of chromosomal, gonadal and anatomical sex, in which results a wide range of phenotypes related to genetic variations, gonadal/genital developmental programming and endocrine system (Hughes et al., 2006; Ostrer, 2014). Although, there is very limited data available on the exact prevalence of DSD, the estimated incidence ranges from 1 per 4.500–5.500 live births for strictly defined “ambiguous genitalia” in European countries to 1 per 2.500–3.000 in certain Arabic communities, due to consanguinity (Bashamboo and McElreavey, 2014). The molecular diagnosis of DSD has been a long-standing challenge and until recently, a genetic diagnosis was available for a small minority of individuals with DSD (Arboleda et al., 2013). Providing an accurate genetic diagnosis can provide essential counselling and guidance for clinical management, notably for the risk of malignancy (Jørgensen et al., 2015). Next generation sequencing (NGS) technologies have improved the diagnostic yield in congenital anomalies including DSD. Targeted sequencing, using panels with selected DSD genes, is reported to have a diagnostic yield of between 30 and 47% in 46, XY DSD cases (Buonocore et al., 2019; Hughes et al., 2019; Xu et al., 2019). Using only the WES approach, Abualsaud et al., 2020, identified potentially causal genetic variants in 42 genes carried by 51% (76/149) of the patient cohort (Abualsaud et al., 2020).

The diagnostic yield of DSD should continue to improve as new genetic factors involved the development of the human gonad are identified. In the last 5 years, exome and genome sequencing in a research environment has lead to the discovery new genes causing both syndromic and non-syndromic DSD forms of both 46, XY and 46, XX DSD. These include the *DHX37, HHAT, LHX9, MYRF, NR2F2, PBX1, PPP2R3C, SOX8,* and *ZNRF3* genes (McElreavey and Bashamboo, 2021). The phenotypic spectrum associated with well-characterised 46, XY DSD genes such as *NR5A1* and *WT1* has also expanded to include 46, XX DSD (McElreavey and Bashamboo, 2021). This suggests that WES should be the method of choice to determine the genetic etiology in DSD.

Here, we performed exome sequencing on a large Algerian cohort of 125 individuals who presented with either syndromic or non-syndromic DSD. The primary goal was to determine the genetic etiology in a North African population, which has a high degree of consanguinity, and that has not been explored at a molecular level for the causes of DSD. The genetic etiology was established in 49.6% (62/125) individuals. Pathogenic variants in the *AR, HSD17B3, NR5A1* and *SRD5A2* genes were the most common causes of 46, XY DSD explaining 13.9% of this sub-cohort. Other variants were identified in genes know to be associated with congenital hypogonadotropic hypogonadism (CHH), including the *CHD7* and *PROKR2* genes. Several variants were in more than one affected individual consistent with genetic founder effects. We found that 11.5% of all 46, XY DSD individuals carried more than one variant classified as pathogenic (P) or likely pathogenic (LP) suggesting that digenic/oligogenic inheritance may be relatively common. This analysis also identified possible new genes involved in DSD as well as atypical clinical presentations.

## Materials and Methods

### Patients and Methods

A cohort of 125 patients with DSD of unknown etiology, referred to clinic pediatric, pediatric surgery and endocrinology centers in eastern/central Algeria, over a period of 2 years (2018–2019), including five familial cases, and one pair of monozygotic twins discordant for DSD were included in the study. The preliminary diagnosis of DSD was based on clinical features of the external genitalia, imaging examinations (abdominal and pelvic ultrasound and magnetic resonance imaging), gonadal histology, hormonal evaluation and karyotyping. Although the initial diagnosis was 46, XY DSD, further hormonal and genetic analysis indicated that some cases were CHH. Genetic sex in each individual was determined by a standard karyotype analysis and confirmed by PCR amplification of *SRY* gene sequences. The cohort consists of five categories:15 patients with 46, XY gonadal dysgenesis/testicular regression sequence (TRS), 1 patient with 46, XY persistent Müllerian duct syndrome (PMDS), 67 patients defined as 46, XY DSD with atypical external genitalia, 39 patients with syndromic 46, XY DSD, and 3 patients with 46, XX testicular/ovotesticular DSD (*SRY*-negative). Patients with 46, XX congenital adrenal hyperplasia (CAH) as well as those with sex chromosomal disorders were excluded. 46, XY testicular regression syndrome is defined by a 46, XY chromosome complement, ambiguous or atypical external genitalia, anomalies of sexual duct formation, and absence of gonadal tissue on one or both sides. Testicular determination is considered to have occurred in boys with TRS but the tissue disappeared before the 16th week gestation when testis formation is complete. All patients (34 families included, according to the phenotype complexity) were screened for variants in genes known to cause DSD by analysis of exome datasets and confirmed by Sanger sequencing of candidate genes. All patients met the revised criteria of the Pediatric Endocrine Society (LWPES)/European Society for Paediatric Endocrinology (ESPE) (Hughes et al., 2006). This study was approved by the local French ethical committee (2014/18NICB—registration number IRB00003835) and consent to genetic testing was obtained from adult probands or from the parents when the patient was under 18 years.

### Exome Sequencing

WES was performed in 125 patients (including 34 families) who presented with anomalies of gonad/genital formation and differentiation. Exonic and adjacent intronic sequences were enriched from genomic DNA using Agilent SureSelect Human All Exon V4, and paired-end sequencing on the Illumina HiSeq2000 platform with TruSeq v3 chemistry. Data analysis was generated from the sequencing platform using manufacturer’s proprietary software. Reads were mapped against the human reference genome (NCBI, GRCh37/hg19 or GRCh38/hg38) *via* Burrows-Wheeler aligner. Single-nucleotide variants and small insertions and deletions (InDel) were carried out with GATK version 1.6. For each patient, duplicate reads and BAM files manipulations were selected by Picard version 1.62 (http://broadinstitute.github.io/picard/), and SAMtools version 0.1.18, respectively. Single-nucleotide polymorphism (SNP) and InDel variants were annotated to dbSNP 138 identifiers using the Genome Analysis Toolkit (GATK) Unified Genotyper. The SNP Effect Predictor bioinformatics tools on the Ensembl website (http://www.ensembl.org/homosapiens/userdata/uploadvariations), gnomAD (https://gnomad.broadinstitute.org/) and ClinVar (https://www.ncbi.nlm.nih.gov/clinvar/) were used to annotated the novel variants, followed by manual screening of all variants by using the Human Gene Mutation Database Professional Biobase (http://www.biobaseinternational.com/product/hgmd/). To determine if rare/novel variants were associated with DSD or any of the somatic feature of the phenotype in the syndromic forms of DSD, we screened all variants against currently available data in OMIM, Pubmed, ClinVar, Orphanet etc, using the online tool VarElect (https://ve.genecards.org/). This tool prioritizes variants related to the phenotype. Each of these genes/variants was then verified manually be checking the appropriate literature in Pubmed. Clinical significance was established according to the 2015 American College of Medical Genetics and Genomics and Association for Molecular Pathology (ACMG) (Richards et al., 2015). Variants that were linked to the pathologies were confirmed with classic Sanger sequencing.

## Results

### Overview of a Large Cohort of DSD patients

**FIGURE 1:**
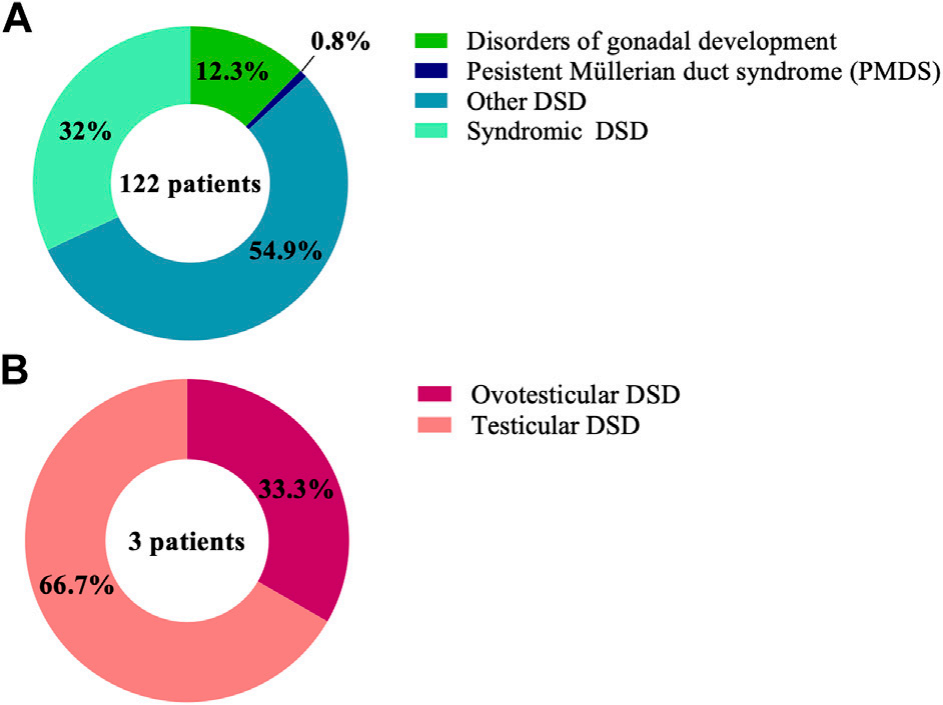
Proportions of the DSD phenotypes in the cohort of 125 patient with **(A)** 46, XY DSD and **(B)** 46, XX DSD.

WES was performed on a large cohort of 125 patients with a wide spectrum of DSD. All individuals were of Algerian ancestry and, for 34 of these patients, additional family members were available for study (Table 1). The age at diagnosis ranged from 1 day to 22 years of age and a history of parental consanguinity was reported in 36% (45/125) of the cohort. The majority of patients (97.6%, 122/125) were diagnosed with 46, XY DSD and of these 13.1% (16/122) were raised as female. 2.4% of patients (3/125) were diagnosed with 46, XX DSD and raised as male. These groups were subdivided in 5 main categories of DSD according to the 2006 Consensus Statement on Management of Intersex Disorders (Hughes et al., 2006) and the proportion of phenotypes are illustrated in Figure 1. Details of the clinical, hormonal and molecular findings for each patient are summarized in Tables 2, 3 and Supplementary Tables S1, S2. Overall 12% (15/125) of patients were classified with 46, XY disorders of gonadal development, where gonadal agenesis or complete/partial gonadal dysgenesis was diagnosed based on the hormonal profiles and/or the gonadal phenotype (Table 2). One patient was diagnosed with 46, XY persistent Müllerian duct syndrome (Table 3) and 53.6% (67/125) of individuals with 46, XY atypical external genitalia were classified as « other DSD ». The “other DSD” subgroup included patients who are considered to have a potential disorder in androgen synthesis or action (DASA) or with unexplained undervirilization (Supplementary Table S1). Syndromic DSD cases represent 31.2% (39/125) of the entire cohort. This category includes subjects diagnosed with 46, XY DSD associated with syndromic obesity or a range of dysmorphic features (Supplementary Table S2). 46, XX DSD consists of individuals with testicular or ovotesticular DSD. Individuals with suspected or confirmed CAH as well as individuals with chromosomal anomalies were not included in the study.

**TABLE 1:** Summary of DNA samples according to relevant phenotypes in a cohort of 122 patients with 46, XY DSD and 3 patients with 46, XX DSD, and their families.

Cohort classification	DNA Samples Collected					WES Analysis			
	Singletons	Duos	Trios	Trios and Siblings	Total	Singletons	Duos	Trios	Trios and Siblings
46, XY DSD									
1. Disorders of gonadal development	2	2	5	6	15	9	1	3	2
2. Persistent Müllerian duct syndrome	0	0	1	0	1	0	0	1	0
3. Other DSD	17	9	31	10	67	55	0	8	4
4. Syndromic DSD	5	8	18	8	39	27	1	9	2
Total	**24**	**19**	**55**	**24**	**122**	**91**	**2**	**21**	**8**
46, XX DSD									
1. Ovotesticular DSD	0	0	0	1	1	0	0	1	0
2. Testicular DSD	0	0	0	2	2	0	0	0	2
Total	**0**	**0**	**0**	**3**	**3**	**0**	**0**	**1**	**2**
Overall Total					**125**	**91**	**2**	**22**	**10**

a Singletons represent affected individual only. Duos represent affected individual and one parent. Trios represent affected individual and both parents. Trios and sibling(s) represent affected individual, both parents and one sibling at least.

**TABLE 2:** Clinical phenotype, hormonal profile and details of variants identified in 12 individuals with disorders of gonadal development.

Case, Age, Sex rearing	FSH (UI/L)	LH (UI/L)	Testosterone (ng/ml)		AMH (ng/ml)	Genitourinary				Variant	MAF and Population (gnomAD)/Predicted effect on Protein	Zyg	PoGI/transmission of Variant	Clinical Significance: ACMG/ClinVar/GV, ref
	Value/NR	Value/NR	Value/NR	Value-HCG Stimulation test/NR	Value/NR	External genitalia	Internal genitalia	Gonadal Position	Gonad/histology					
1 2D M	NA	NA	0.08 [0.12–0.21] (3D)	NA	NA	Phallus (<1 cm), BC, NPG	No Müllerian ducts, posterior cavity present, male urethra	No residual gonad	No residual gonad	DHH:NM_021044:c.G913A:p.G305R	Novel/SIFT: tolerated (0.14), PolyPhen2: possibly damaging (0.993), REVEL: 0.528 (LDC)	Het	AR Paternal	VUS/NA/NA
										GPRC6A:NM_148963:c.2323dupT:p.Y775fs	0.2768- African/African-American/LOF	Het	Maternal	B/B/VUS Dawson et al. (2020)
2 2D M	NA	NA	0.09 [<0.03] (14D)	<0.025 [0.03–0.38] (3.5Y)	0.01 [1.0–10.6] (3Mo)	Micropenis (1 × 0.5 cm), urogenital sinus closed, urethral orifice, hypoplastic scrotum, BC, NPG	No Müllerian ducts	R: inguinal, L: inguinal	R:hypotrophic hypoechoic testis (10.6 × 4mm)/NA, L: testis (11 × 6mm)/NA	PROK2:NM_001126128:c.96+4A > G	Novel/LOF	Het	AD	VUS/NA/NA
3 40D F	NA	NA	<0.03 [0.03–0.32] (4Y)	NA	1.44 [12.6–167] (4.5Y)	Micropenis (3 cm), perineal hypospadias, labia majora, BC, NGP	Hypoplastic uterus, Müllerian ducts present	R: abdominal, L: abdominal	R: ovary/NA, L: ovary/NA	RNF216:NM_207111:c.G785A:p.R262H	0.0005534- Other/SIFT: tolerated low confidence (0.05), PolyPhen2: B (0.169), REVEL: 0.016 (B)	Het	AR	VUS/NA/NA
4 3Y M	2.9 [1–8] (3Y)	0.2 [0.6–12] (3Y)	0.026 [3.0–12.0] (3Y)	0.34 [3.0–12.0] (3Y)	0.08 [3.8–159.8] (3Y)	Genital bud (4 cm), posterior orifice, poorly developed labioscrotal folds, BC, NPG	No Müllerian ducts	R: abdominal, L: no residual gonad	R: nodular structure (11 × 17 mm)/NA, L: no residual gonad	ANOS1:NM_000216:c.C1187T:p.S396L	0.003935- Ashkenazi Jewish/SIFT: deleterious (0), PolyPhen2: B (0.085), REVEL: 0.462 (B)	Hem	XLR	VUS/P/LP Dodé et al. (2006)
										WT1:NM_024424:c.C299G:p.A100G	Novel/SIFT: tolerated low confidence (0.59), PolyPhen2: B (0.007), REVEL: 0.219 (B)	Het	AD Parental	LB/NA/B McCarthy et al. (2013)
5 # 1D M	4.33 [<5.0] (6Mo)	1.09 [<1.0] (6Mo)	0.12 [0.12–0.22] (6Mo)	2.57 [0.12–0.22] (6Mo)	67.6 [39.1–91.1] (6Mo)	Micropenis (2 cm), 2 orifices, anogenital distance of 4cm, PG	Müllerian ducts present, oblong posterior cavity (18 mm) communicating with the bulbar urethra	R: inguinal, L: inguinal	R:hypotrophic testis/NA, L: hypotrophic testis/NA	NR5A1:NM_004959:c.A1223C:p.H408P	Novel/SIFT: tolerated (0.24), PolyPhen2: possibly damaging (0.641), REVEL: 0.769 (LDC)	Het	AD *De novo*	P/NA/NA
										MAP3K1:NM_005921:c.A3418G:p.M1140V	0.001024- East Asian/SIFT: deleterious (0.04), PolyPhen2: B (0.01), REVEL: 0.180 (B)	Het	AD	VUS/NA/NA
										CTU2:NM_001012759:c.C710T:p.A237V	0.0009344- European (Finnish)/SIFT: deleterious (0), PolyPhen2: possibly damaging (0.581), REVEL: 0.184 (B)	Het	AD	VUS/NA/NA
6 # 45D M	8.88 [1.1–25] (2Mo)	0.2 [1.5–11.8] (2Mo)	1.70 [2.2–10.5] (2D)	NA	<0.03 [42–203] (3Mo)	Micropenis (<0.5 cm), posterior hypospadia, hypoplastic labia, BC, NPG	Müllerian ductspresent	No residual gonad	No residual gonad	DHX37:NM_032656:c.G923A:p.R308Q	Novel/SIFT: deleterious (0), PolyPhen2: probably damaging (1), REVEL: 0.451 (B)	Het	AD *De novo*	P/NA/P McElreavey et al. (2020)
										GLI2:NM_005270:c.C1289G:p.A430G	Novel/SIFT: deleterious (0.01), Polyphen2: B (0.205), REVEL: 0.095 (B)	Het	AD	VUS/NA/NA
										CCDC141:NM_173648:c.C3782T:p.A1261V	0.0003274- Other/SIFT: tolerated (0.26), PolyPhen2: B (0.029), REVEL: 0.056 (B)	Het	AR	VUS/NA/NA
7 2D M	NA	NA	<0.10 [3.0–10.6] (18Mo)	NA	46.3 [51.3–88.3] (22Mo)	Genital bud (2 cm), perineal hypospadias, bifid scrotum, BC, NPLG	Uterus remnant present, uterovaginal cavity present	R: inguinal, L: no residual gonad	R: testis/NA, L: no residual gonad	MYRF:NM_001127392:c.A1222G:p.I408V	0.001779- Latino/Admixed American/SIFT: tolerated (0.19), PolyPhen2: B (0.241), REVEL score: 0.062 (B)	Het	AD Maternal	VUS/NA/NA
										CCDC141:NM_173648:c.G1131T:p.K377N	0.001264- Ashkenazi Jewish/SIFT: deleterious (0), PolyPhen2: probably damaging (0.982), REVEL: 0.110 (B)	Het	AR Maternal	VUS/NA/NA
										TGIF1:NM_170695:c.239dupC:p.A80fs	Novel/LOF	Het	AD Maternal	VUS/NA/NA
8 # 20Mo M	86.76 (6Mo)	11.38 (6Mo)	0.03 [3.0–12.0] (6Mo)	0.17 [3.0–12.0] (6.5Mo)	0.01 [2.0–6.8] (6Mo)	Micropenis (0.5 cm), fused pigmented labia, fusion of labia minora, BC, NPG	No Müllerian ducts	No residual gonad	No residual gonad	DHX37:NM_032656:c.G923A:p.R308Q	Novel/SIFT: deleterious (0), PolyPhen2: probably damaging (1), REVEL: 0.451 (B)	Het	AD Maternal	P/NA/P McElreavey et al. (2020)
										SLC29A3:NM_018344:c.C971T:p.P324L	0.0004349- East Asian/SIFT: deleterious (0), PolyPhen2: probably pathogenic, REVEL: 0.899 (LDC)	Het	AR Paternal	VUS/LP/VUS Molho-Pessach et al. (2014)
										CCDC141:NM_173648:c.G1979A:p.R660Q	Novel/SIFT: deleterious (0.04), PolyPhen2: tolerated (0.121), REVEL: 0.074 (B)	Het	AR Maternal	VUS/NA/NA
9 2D F	NA	NA	0.35 [0–2.31] (2.5Mo)	NA	3.6 [3.8–159.8] (14D)	Clitoromegaly (1.5 cm), bifid poorly developed labiscrotal folds	Presence of a left structure correspond probably to a Müllerian ducts	R: inguinal, L: inguinal	R: testis/NA, L: testis/NA	FGFR2:NM_000141:c.A1132G:p.I378V	Novel/SIFT: tolerated (0.37), PolyPhen2: possibly damaging (0.503), REVEL: 0.290 (B)	Het	AD Paternal	VUS/NA/NA
										FANCD2:NM_033084:c.C2965G:p.P989A	0.0004573- South Asian/SIFT: deleterious (0.02), PolyPhen2: B (0.311), REVEL: 0.091 (B)	Het	AR Paternal	VUS/VUS/NA
10 4.5Mo M	NA	NA	0.1 [3.0–12.0] (2.5Mo)	0.5 [3.0–12.0] (9Mo)	18.7 [51.3–88.3] (13Mo)	Micropenis (<1 cm), UC	No Müllerian ducts	R: scrotum, L: inguinal	R:oscillating testis/NA, L: testis/NA	RXFP2:NM_130806:c.G184A:p.A62T	0.001524- Other/SIFT: tolerated (0.43), PolyPhen2: B (0.035), SIFT: 0.281 (B)	Het	AR	VUS/NA/NA
12 30Mo M	NA	NA	NA	0.639 [0.0–0.9] (30Mo)	116.0 [3.8–159.8] (30Mo)	Micropenis (1 cm), phimosis, urethral orifice, developed scrotum, UC	No Müllerian ducts	R: scrotum, L: no residual gonad	R: testis (15 × 9 mm)/NA, L: no residual gonad	MAP3K1:NM_005921:c.A3557G:p.E1186G	Novel/SIFT: deleterious (0.01), Polyphen2: probably damaging (0.986), REVEL: 0.259 (B)	Het	AD	VUS/NA/NA
										MYRF:NM_001127392:c.A1222G:p.I408V	0.001779- Latino/Admixed American/SIFT: tolerated (0.19), PolyPhen2: B (0.241), REVEL: 0.062 (B)	Het	AD Maternal	VUS/NA/NA
15 22Y M	69.26 [1–8] (22Y)	36.36 [2–12] (22Y)	0.32 [3–10.6] (22Y)	0.34 [0.7–8.53] (22Y)	0.04 [0.8–14.6] (22Y)	Penis (5 cm), urogenital sinus closed, urethral orifice, developed scrotum, BC, NPG	No Müllerian ducts, atrophic prostate present	No residual gonad	No residual gonad	SOX8:NM_014587:c.G1264A:p.G422S	0.0001749- Other/SIFT: tolerated (1), PolyPhen2: B (0.003), REVEL: 0.194 (B)	Het	AD	VUS/NA/NA
										PROKR2:NM_144773:c.C868T:p.P290S	0.0003668- Latino/Admixed American/SIFT: deleterious (0), PolyPhen2: probably damaging (1), REVEL: 0.939 (LDC)	Het	AD	VUS/VUS/VUS Cox et al. (2018)
										PLXNA3:NM_017514:c.787_796del:p.V263fs	Novel/LOF	Hem	AR	VUS/NA/NA
										FLNA:NM_001110556:c.C2449T:p.P817S	Novel/SIFT: deleterious (0.02), PolyPhen2: B (0.439), REVEL: 0.320 (B)	Hem	XLD	VUS/NA/NA
										NIPBL:NM_015384:c.6954+3A > G	0.0001693- African/African-American/LOF	Het	AD	VUS/NA/NA
										SLC29A3:NM_001174098:c.G325A:p.V109I	0.0003252- European (non-Finnish)/SIFT: tolerated (1), PolyPhen2: B (0.007), REVEL: 0.052 (B)	Het	AR	VUS/VUS/NA
										GLI3:NM_000168:c.G1527C:p.E509D	Novel/SIFT: deleterious (0.02), PolyPhen2: B (0.038), REVEL: 0.122 (B)	Het	AD	VUS/NA/NA

ACMG, american college of medical genetics; AD, autosomal dominant; AMH, anti-Müllerian hormone; AR, autosomal recessive, B benign, BC, bilateral cryptorchidism, D day, DSD, disorders/differences of sex development, F female, FSH, follicle stimulating hormone; GD, gonadal dysgenesis, gnomAD genome aggregation database; GV, gene variants previously associated with the disease; HCG, human chorionic gonadotropins, Hem hemizygous, Het heterozygous, Hom homozygous; I.D., initial diagnosis, L left, LB, likely benign; LDC, likely disease causing; LH, luteinizing hormone; LOF, loss-of-function, LP, likely pathogenic; LPG, left palpable gonad, M male, MAF, minor allele frequency, Mo month, NA not available; NPG, non palpable gonads; NPLG, non palpable left gonad; NPRG, non palpable right gonad; NR, normal range, P pathogenic, PG, palpable gonads; PGD, partial gonadal dysgenesis, PoGI, pattern of disease inheritance usually associated with the gene, R right, Ref reference, REVEL, rare exome variant ensemble learner; RPG, right palpable gonad; TRS, testicular regression syndrome; UC, unilateral cryptorchidism; VUS, variant of uncertain significance, Y year. ^#^A definitive genetic diagnosis was achieved.

**TABLE 3:** Clinical phenotype, hormonal profile and details of variants identified in 1 individual with 46, XY persistent Müllerian duct syndrome (PMDS).

Case Age Sex of rearing	FSH (UI/L)	LH (UI/L)	Testosterone (ng/ml)		AMH (ng/ml)	Genitourinary				Variant	MAF and Population (gnomAD)/Predicted effect on Protein	Zyg	PoGI/transmission of Variant	Clinical Significance: ACMG/ClinVar/GV
	Value/NR	Value/NR	Value/NR	Value-HCG Stimulation test/NR	Value/NR	Externalgenitalia	Internal genitalia	Gonadal Position	Gonad/histology					
16 # 18Mo M	NA	NA	NA	8.28 [0.42–0.8] (19Mo)	0.01 [21–210] (17Mo)	Developed phallus, scrotum, BC	Müllerian Ducts present	R: abdominal cavity, L: abdominal cavity	R: testis/Testicularparenchyma, seminiferous tubules with immature germcells, L: testis/NA	AMH:NM_000479:c.127_128del:p.L43fs	Novel/LOF	Hom	AR	P/NA/NA
										GPRC6A:NM_148963:c.T1969C:p.F657L	0.0001386- Other/SIFT: tolerated (0.34), PolyPhen2: B (0.079), REVEL: 0.430 (B)	Het	Paternal	LB/NA/NA

ACMG American College of Medical Genetics, AD autosomal dominant, AMH anti-Müllerian hormone, AR autosomal recessive, B benign, BC bilateral cryptorchidism, FSH follicle stimulating hormone, gnomAD genome aggregation database, GV gene variants previously associated with the disease, Het heterozygous, HCG human chorionic gonadotropins, Hom homozygous, L left, LB likely benign, LH luteinizing hormone, LOF loss-of-function, M male, MAF minor allele frequency, Mo month, NA not available, NR normal range, P pathogenic, PMDS persistent Müllerian duct syndrome, PoGI Pattern of disease inheritance usually associated with the gene, R right, LB likely benign, Ref reference, REVEL rare exome variant ensemble learner. ^#^A definitive genetic diagnosis was achieved.

### WES Analysis and Evaluation of Pathogenicity

**FIGURE 2:**
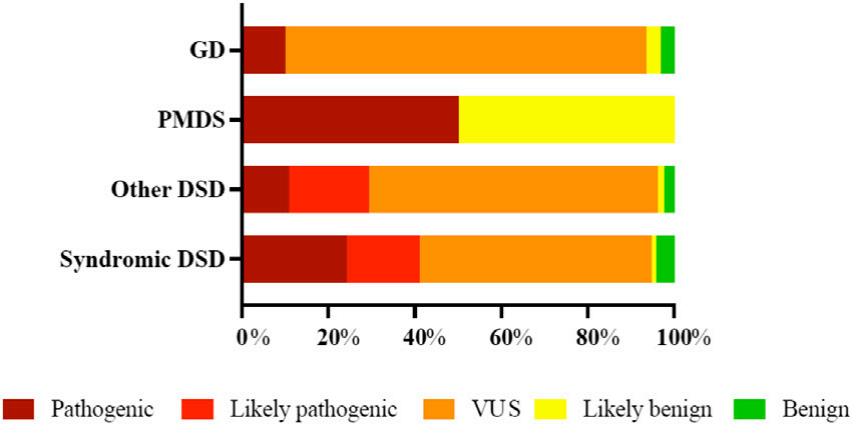
Distribution and percentage of curated 258 variants at different evidence levels in the fourth categories of patients with 46, XY DSD as defined in Table 4. The percentages refer to only to the variants that are classified in each patient category. * DSD, disorders of sex development, GD, gonadal dysgenesis, PMDS, persistent Müllerian duct syndrome, VUS, variant of uncertain significance.

**FIGURE 3:**
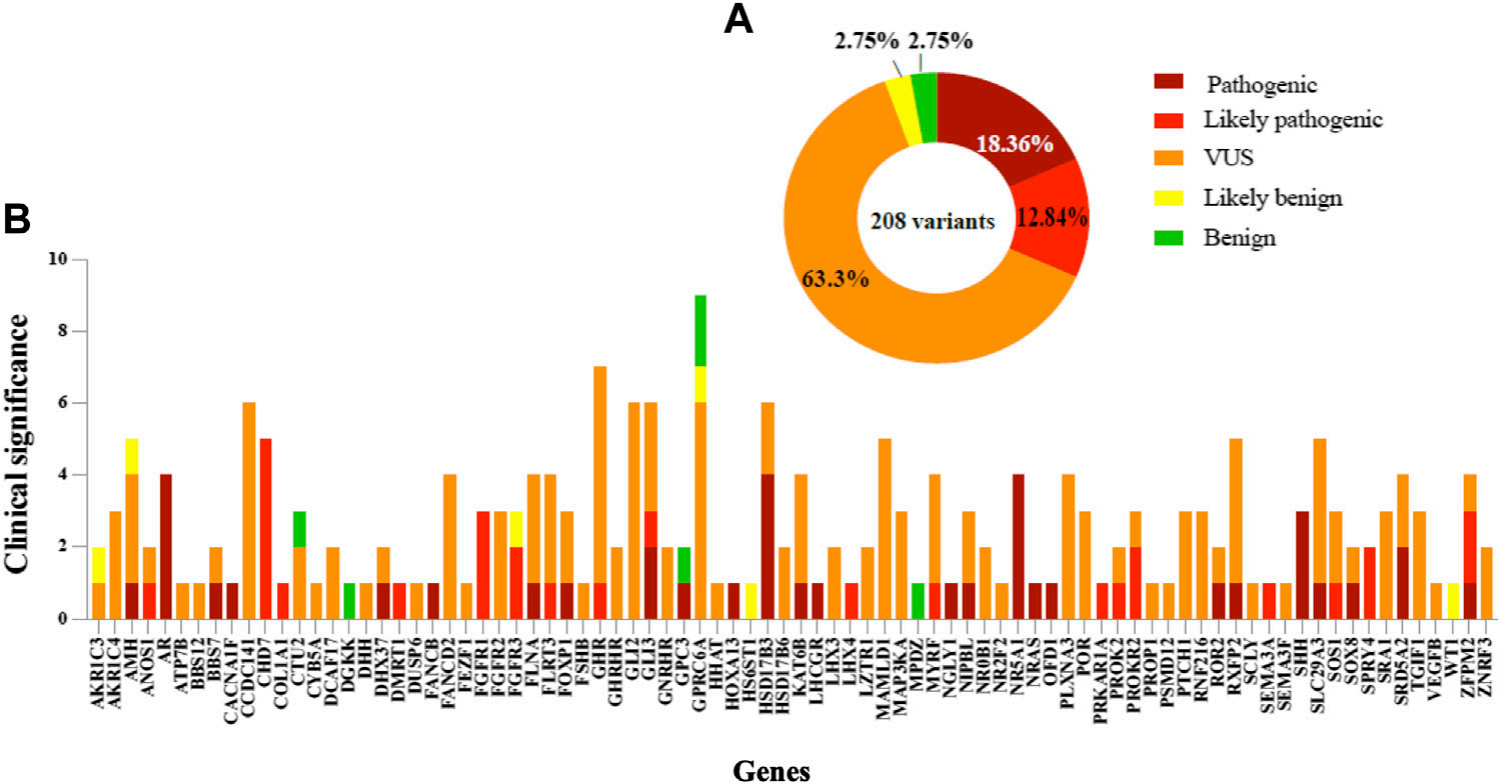
Distribution and classification variants in DSD cohort. **(A)** Summary of 208 variants frequency found for each classification. **(B)** Variants in 84 genes in which the genetic cause was identified in syndromic/non-syndromic 46, XY DSD. The pathogenicity of variants is shown for each gene.

In accordance with the 2015 American College of Medical Genetics and Genomics and Association for Molecular Pathology (ACMG) guidelines, variant proportions at different evidence levels of pathogenicity in the cohort are illustrated in Figure 2. P/LP variants were identified in 49.6% (62/125) of all cases (Table 4) in 42 genes (Figure 3). Overall four individuals carried novel or rare heterozygous P variants in the *NR5A1* gene (DSD cases 5,72, 86, and 87), whilst four other individuals carried novel hemizygous P variants in the *AR* gene (DSD cases 43, 63, 98, and 106). We also identified 4 novel or rare and homozygous P variants in the *HSD17B3* gene, which were carried by 5 individuals (DSD cases 20, 38, 50, 78, and 93). Four other individuals carried 2 rare homozygous P variants in *SRD5A2* gene (DSD cases 19, 34, 40, and 57). Five novel or rare heterozygous LP variants were identified in *CHD7* gene in 7 cases (DSD cases 25,41, 45, 62, 75, 97, and 112) and in the *PROKR2* gene 2 rare and LP variants were carried by 8 cases (DSD cases 29, 45, 57, 62, 66, 67, 87, and 102). Potentially P/LP variants were confirmed by Sanger sequencing in patients, as well as available parents and siblings. However, no causative variants were identified in the three patients with 46, XX DSD nor in 18 individuals with 46, XY DSD including 4 patients from two families.

**TABLE 4:** Distribution and characteristics of pathogenicity in curated 256 variants in cohort of 125 patients with syndromic/non-syndromic DSD phenotypes.

Cohort classification	Patients grouped according to variant class			Number of variants					Total
	Patients with no variants	Patients with VUS/LB/B variants	Patients with P/LP variants	B	LB	VUS	LP	P	
46, XY DSD									
1. Disorders of gonadal development	3	9	3	1	1	25	0	3	30
2. Persistent Müllerian duct syndrome	0	0	1	0	1	0	0	1	2
3. Other DSD	11	25	31	3	2	87	24	14	130
4. Syndromic DSD	4	8	27	4	1	52	15	23	95
Total	**18**	**42**	**62**	8	5	164	39	41	**257**
46, XX DSD									
1. Ovotesticular DSD	1	0	0	0	0	0	0	0	0
2. Testicular DSD	2	0	0	0	0	0	0	0	0
Total	**3**	**0**	**0**	0	0	0	0	0	**0**
Overall Total				8	5	164	39	41	**257**

Several potentially pathogenic variants were observed in more than one affected individual. This includes the known pathogenic p.R85C amino acid substitution in the PROKR2 protein (McCormack et al., 2017). This variant was identified in 4 unrelated individuals (DSD cases 29, 62, 87, and 102) and 2 brothers (DSD cases 66 and 67) with atypical external genitalia. In one of these individuals the variant was *de novo* (DSD case 29). In the other 5 individuals the parental origin is unknown. A second substitution (p.P290S) in the same protein was reported in the literature as a VUS (Cox et al., 2018) and probably pathogenic in 2 unrelated cases (DSD cases45 and 57) diagnosed with androgen synthesis/action disorders or SRD5A2 deficiency. This variant was inherited from the mother in one case (DSD case 45) and in the second one (DSD case 57), the inheritance is unknown. A rare and previously reported P variant (p.Y235F) in *SRD5A2* was carried in the homozygous state by 3 apparently unrelated individuals (DSD cases 19, 34, and 57), who presented with DASA. One novel heterozygous variant (p.R308Q) in the *DHX37* gene, previously described as pathogenic (McElreavey et al., 2020) was carried by 2 unrelated individuals (DSD cases 6 and 8), explaining their TRS phenotype. In one of these individuals the variant was *de novo* and in a second it was inherited from the mother (Zidoune et al., 2021).

Overall, 11.5% (14/122) of all 46, XY DSD patients were found to carry P/LP variants in more than one gene known to be associated with DSD (*ANOS1, AR, BBS7,CHD7, FGFR1, FGFR3, FLNA, GPC3, HOXA13, KAT6B, LHCGR, NRAS, NR5A1, OFD1, PRKAR1A, PROK2, PROKR2, RXFP2, SEMA3A, SHH* and *SRD5A2*). This includes both syndromic (8/122) and non-syndromic (6/122) 46, XY DSD cases.

### 46, XY DSD Associated With Genes Known to Cause CHH

A total of 43 rare or novel variants were found in 20 genes associated with congenital hypogonadotropic hypogonadism CHH (*ANOS1*, *CCDC141*, *CHD7, DUSP6, FEZF1, FGFR1, FLRT3, FSHB, GNRHR, HS6ST1,LHX3, LHX4, PLXNA3, PROK2, PROKR2, PROP1, RNF216, SEMA3A, SEMA3F* and *SPRY4*). These variants were carried by 36% (45/125) of the entire cohort. Of these variants, 17 were classified as P/LP involving the *ANOS1*, *CHD7, FGFR1, FLRT3, LHX4, PROK2, PROKR2, SEMA3A* and *SPRY4* genes (Figure 3). Pathogenic variants in these genes cause CHH, a rare genetic condition due to inadequate hypothalamic gonadotropic-releasing hormone (GnRH) axis activation or a failure of pituitary gonadotropin secretion (Stamou and Georgopoulos, 2018). The P/LP variants are harbored by 16.8% (21/125) of the cohort (DSD cases 23–25, 29, 36, 41, 45, 57, 62, 66–68, 75, 81, 86, 87, 89, 97, 98, 102 and112). Of these, one patient (DSD 62) with atypical external genitalia and a positive HCG response carried 3 different missense variants in the *ANOS1*, *CHD7* and *PROKR2* genes. Another patient (DSD 45) carried 2 missense variants in *CHD7* and *PROKR2* genes and presented with undervirilized external genitalia and normal response to HCG stimulation, and one patient (DSD 25) carried 2 missense variants in *CHD7* and *SEMA3A* genes and presented with micropenis, hypospadias, and bilateral cryptorchidism.

Overall, 8 patients (DSD cases 23, 36, 45, 62, 68, 98, 102 and 112) harbored P/LP variants in the *ANOS1, CHD7, FGFR1, LHX4, PROKR2* and *SPRY4* genes. These individuals showed a positive HCG response and had normal male levels of AMH, suggesting a diagnosis of CHH. However, there are atypical cases. DSD 86 carried a *de novo* pathogenic *NR5A1* variant as well as likely pathogenic variant in *PROK2*. The phenotype is consistent with a contribution of both of these genes to the pathology. The gonadotropin levels were low as well as low levels of both testosterone and AMH. Recently, LOF variants in two genes, *SEMA3F* and its receptor *PLXNA3* have been proposed as a cause of CHH (Kotan et al., 2021). Rare and novel variants in both of these genes were identified in 5 individuals (DSD cases 15, 31, 36, 39 and 86). One case (DSD 15) carries a frameshift variant in *PLXNA3* (p.V263fs). The *PLXNA3* gene is located on the X chromosome and this variant is predicted to be LOF. This 22 year old male presented with gonadal dysgenesis/TRS and no residual gonadal material was found. Consistent with the absence of the testis, but inconsistent with a role of PLXNA3 in controlling gonadotropin production, the levels of gonadotropins were highly elevated (Table 2).

### Novel Genetic variants Associated With DSD

**FIGURE 4:**
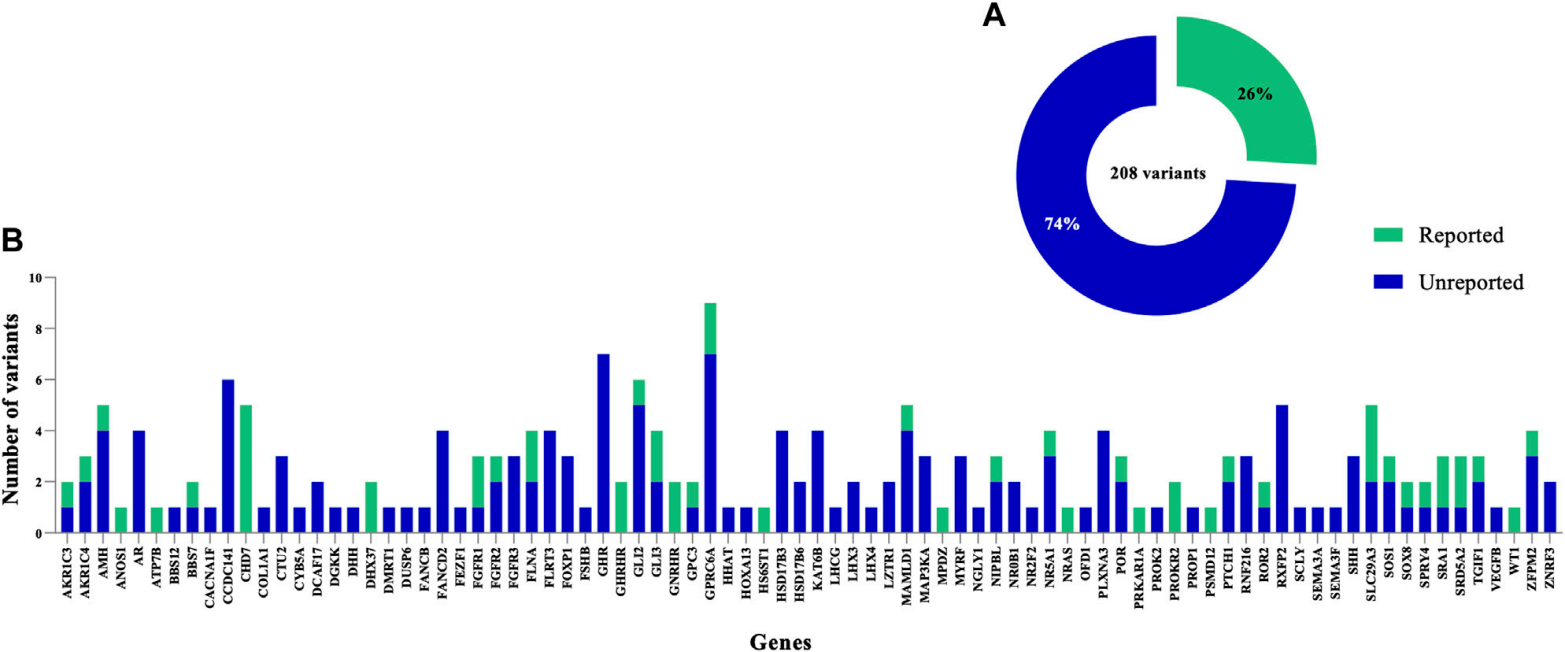
Distribution of previously reported and novel genetic variants identified in the DSD cohort. **(A)** Summary indicating the proportion of previously reported variants. **(B)** The clinical significance of variants in 84 genes identified in this study among the syndromic/non-syndromic 46, XY DSD cohort. The total number of variants is shown for each gene, including previously reported variants and novel ones.

In this study, only 26% (54/208) of the total variants identified in known DSD genes were previously reported in the literature (Figure 4). Of these 54 variants, 22 were previously published and classified as P/LP in the*ANOS1, CHD7, DHX37, FGFR1, FLNA, GLI3, NR5A1, NRAS, PRKAR1A, PROKR2, SLC29A3, SOS1, SPRY4, SRD5A2* and *ZFPM2* genes, and these were carried by a total of 27 patients. Overall, the most common was a LP variant (p.R85C) in the *PROKR2* gene, which was carried by 6 boys with atypical external genitalia. A second likely pathogenic variant (p.P290S) in PROKR2, was carried by 2 unrelated patients (DSD case 45, raised as male and DSD case 57, raised as female), who were initially diagnosed with PAIS or SRD5A2 deficiency. Furthermore, a rare, homozygous and pathogenic variant (p.Y235F) in SRD5A2 was carried by 3 unrelated patients (DSD cases 19, 34 and 57) who presented with an initial diagnosis of PAIS or SRD5A2 deficiency and were raised as female. The CHD7 variant (p.L2806V) was classified as likely pathogenic in 3 unrelated patients (DSD cases 41, 45 and 62) raised as male, and the pathogenic DHX37 variant (p.R308Q) was carried by 2 patients (DSD cases 6 and 8) presented with gonadal dysgenesis and raised as male (Zidoune et al., 2021). A high proportion (74%) of all variants are not reported previously in literature. Of these, 30 were classified as P/LP in 22.4% (28/125) of patients and included a total of 25 different genes. Within this group of genes, 6 carried more than one novel and P/LP variant. These included the *AR* (DSD cases 98, 43, 63 and 106), *FGFR3* (DSD cases 35, 49 and 121), *HSD17B3* (DSD cases 20, 38, 50, 78 and 93), *NR5A1* (DSD case 5, 72 and 86), *SHH* (DSD cases 49, 74 and 118) and *ZFPM2* (DSD cases 64 and 91) genes.

### Genetic variants in 46, XY Non-syndromic DSD

The genetic etiology was established in 42.1% (35/83) of all 46, XY non-syndromic DSD individuals (Tables 2, 3 and Supplementary Table S1). B/LB variants or VUS were carried by 41% (34/83) of patients with 46, XY non-syndromic DSD. In 16.9% (14/83) of patients, variants associated with the phenotypes could not be identified. The most common genetic causes (9.6%; 8/83) of non-syndromic DSD were homozygous or biallelic variants in the *SRD5A2* and *HSD17B3* genes.

Although the aim of this study was not to identify new genetic causes of DSD, the analysis of this group did provide some novel observations that both suggest new genetic causes of 46, XY DSD and also expand the phenotypic spectrum associated with genes known to cause DSD. Heterozygous, loss-of-function variants in the nuclear receptor *NR2F2* have recently been reported in association with a rare syndromic form of 46, XX *SRY*-negative DSD (Bashamboo et al., 2018; Carvalheira et al., 2019). To date, *NR2F2* variants have not been reported in association with 46, XY DSD. We identified a novel *de novo* NR2F2 missense variant of uncertain significance (p.R246H) carried by a 46, XY boy (DSD 37), who presented with micropenis and hypospadias and no other somatic anomalies. This variant is considered likely disease causing according to the REVEL score (0.992). This boy also carried paternally inherited *GLI2* and *GLI3* variants of unknown significance.

**FIGURE 5:**
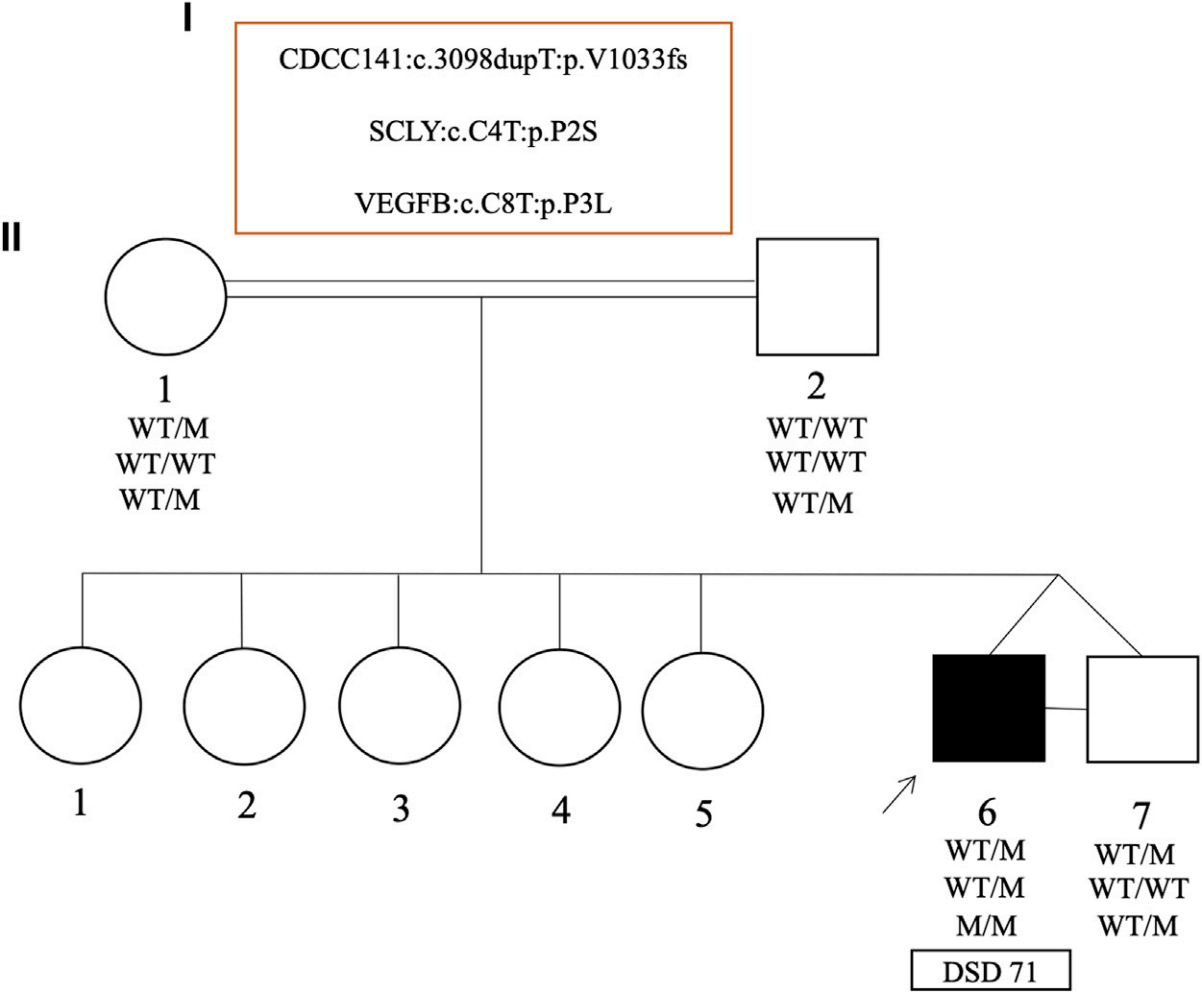
Pedigrees and representative segregation of the curated variants in the monozygotic twins (case 71) discordant for 46, XY DSD. The genotype of family members is indicated. Squares represent male family members and circles represent female family members. Solid squares represent the affected 46, XY subject who was raised as a boy.

In one unusual family with monozygotic 46, XY twins, one twin presented with curved micropenis, penoscrotal hypospadias and undescended hypotrophic testes, where the left testis was non-palpable (DSD 71). Whereas the other twin had typical male genitalia. Both brothers carried a maternally inherited and novel heterozygous frameshift variant (p.V1033fs) in *CCDC141* gene. Biallelic but not monoallelic variants in *CCDC141* are associated with hypogonadotropic hypogonadism (Turan et al., 2017) and hence this heterozygous variant was classified as a VUS. Although the twin boys are monozygotic, they did carry subtle differences in their exome datasets, which were confirmed by Sanger sequencing. The affected twin was homozygous for a novel missense variant (p.P3L) in the *VEGFB* gene, whereas this variant was heterozygous in the unaffected brother, as well as both parents. The affected brother also carried a novel and *de novo* heterozygous missense variant in the *SCLY* gene (p.P2S) that was not present in the unaffected brother (Figure 5).

**FIGURE 6:**
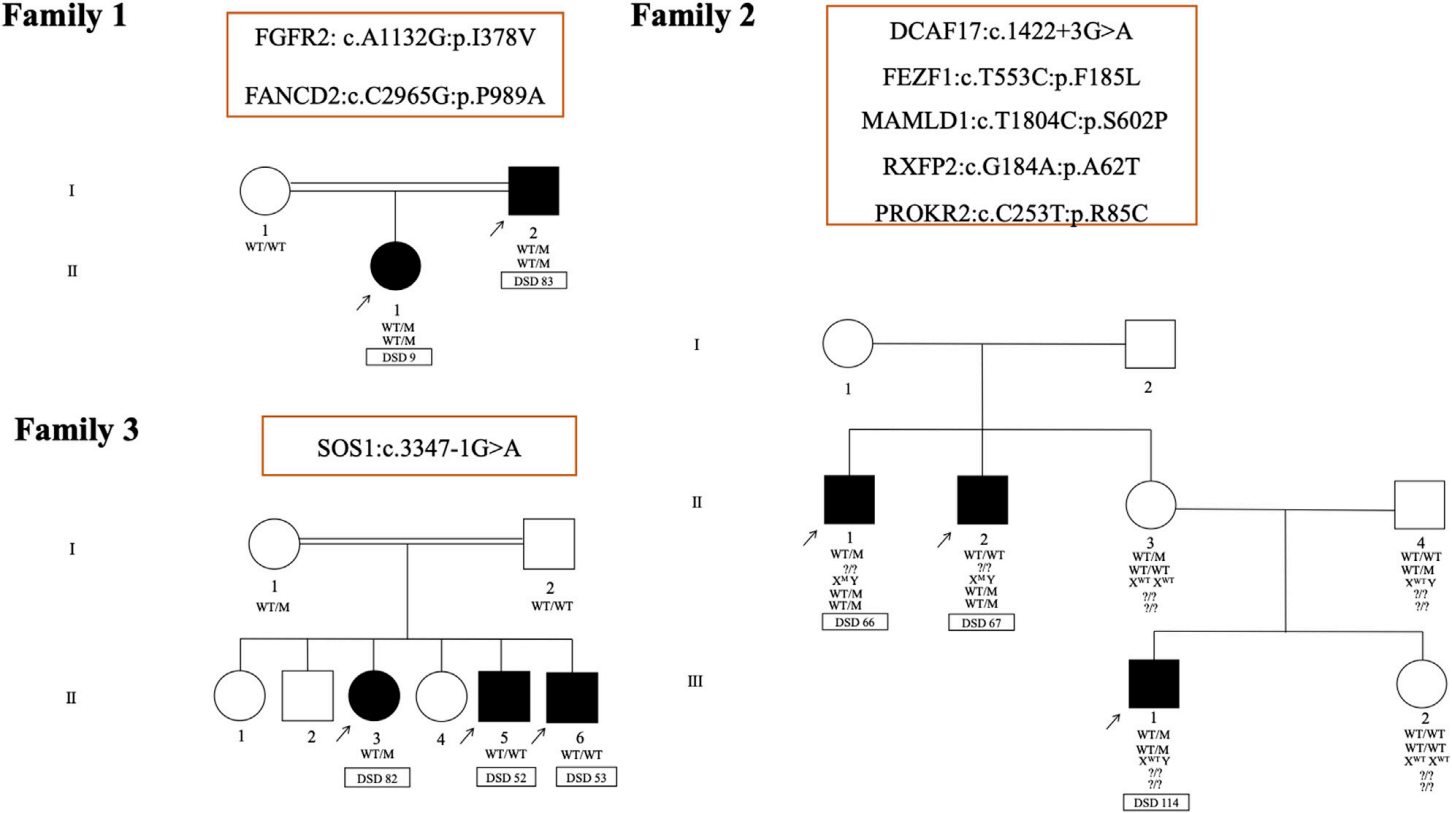
Pedigrees and representative segregation of the curated variants in three unrelated families with 46, XY DSD. Family 1 includes DSD cases 9 and 83. Family 2 includes DSD cases 66, 67 and 114and family 3 includes DSD cases 52, 53 and 82. Genotypes of family members is indicated. Squares represent male family members and circles represent female family members. Solid squares represent affected 46, XY subjects who were raised as boys. Squares containing solid circles represent affected 46, XY subjects who were raised as girls.

The familial occurrence of DSD is considered to be exceptionally rare (Sarafoglou and Ostrer, 2000). Here, in three unrelated families with intrafamilial variability in the expression of DSD, 8 different variants were identified in a total of 8 affected individuals (Figure 6). In the first family (DSD cases 9 and 83), a novel missense variant (p.I378V) in FGFR2 and a rare missense variant (p.P989A) in FANCD2 were identified. Both of these variants are carried by DSD 9 (Table 2) and inherited from her undervirilized father DSD 83 (Supplementary Table S1). The second family (DSD cases 66, 67 and 114) consists of one patient with syndromic DSD (DSD 114) and his two affected maternal uncles, who each presented with micropenis, perineal hypospadias and unilateral cryptorchidism (DSD cases 66 and 67). A total of five variants may contribute to the phenotype in this family. Three missense variants are shared by both brothers (DSD cases 66 and 67) in MAMLD1 (hemizygote, p.S602P), RXFP2 (heterozygous, p.A62T) and PROKR2 (heterozygous, p.R85C). However, their affected nephew (DSD 114) harbored two variants in 2 other genes, a heterozygous missense variant (p.F185L) in FEZF1, which is associated with CHH and was inherited from his healthy father. The second is a heterozygous splice site variant of uncertain significance (c.1422+3G > A) in the*DCAF17* gene. This is also shared by his maternal uncle DSD 66*.*

In the last family of 3 siblings with similar 46, XY DSD phenotypes (DSD cases 52, 53and 82), a heterozygous splice site variant of uncertain significance (c.3347-1G > A) in *SOS1* gene (Noonan syndrome 4; MIM 610733) was identified in the sister (DSD 82). This variant was inherited from the mother and was not transmitted to the brothers (DSD cases 52 and 53). In both brothers, no variants were identified in genes know to be associated with DSD.

### Identification of variants in 46, XY Syndromic DSD

Of 39 patients with 46, XY syndromic DSD, 69.2% (27/39) carried P/LP variants explaining either partially or totally the phenotype of each patient (Supplementary Table S2). P/LP variants were carried by seven individuals (DSD cases 86, 89, 91, 97, 98, 106 and 112), which may explain DSD phenotype but not the somatic features of their phenotypes. These include the genes *AR*, *CHD7, FGFR1, NR5A1, PROK2, PROKR2, RXFP2,* and *ZFPM2*. P/LP variants were carried by 14 subjects (DSD cases 92, 94, 95, 100, 105, 107, 113 and 116–122) carried in genes that are known to cause syndromic forms of DSD including *FANCB, FGFR3, FOXP1, GLI3, GPC3, HOXA13, KAT6B, MYRF, NIPBL, NRAS, OFD1, PRKAR1A, ROR2, SHH, SLC29A3* and *SOS1.* In each of these 14 cases the variants explained partially or totally the phenotype. A further two cases (DSD cases 87 and 102) harbored pathogenic variants in DSD-related genes (*NR5A1* and *PROKR2*) as well as gene variant likely to cause the syndromic form of the phenotype (*BBS7, FGFR3, FLNA* and *GLI3* genes). One subject (DSD 93) harbored a P homozygous variant in *HSD17B3* as well as gene variant likely to cause the syndromic form of the phenotype (*COL1A1* gene; MIM 259420). Two subjects (DSD cases 101 and 104) carried pathogenic variants which explain only the somatic features (*CACNA1F* and *NGLY1*, respectively) of the phenotype but not the DSD. Within this 46, XY DSD subgroup, 20.5% (8/39) carried variants that are either B or VUS. An analysis of the exome datasets did not identify gene variants that may be responsible for the phenotype in 10.3% (4/39) of individuals with these phenotypes.

### 46, XX DSD

WES analysis did not reveal variants linked to DSD in the three cases with 46, XX DSD. The first patient (DSD 123) presented with a curved penis, perineal hypospadias, unilateral cryptorchidism, hypoechoic uterus, vagina and hypotrophic testes. The second patient (DSD 124) presented with 46, XX testicular. A maternal cousin of this patient had hypospadias. The third patient (DSD 125) presented with micropenis, perineal hypospadias, uterus and vaginal cavity. Gonads were palpable in inguinal regions where the right gonad was characterized by ovarian parenchyma with many small follicles and the left gonad was characterized by infantile testicular parenchyma indicating 46XX ovotesticular DSD.

## Discussion

In this DSD cohort of 125 cases of Algerian ancestry, the genetic cause of the phenotype was established in 62 individuals (49.6%). Overall 68 pathogenic or likely pathogenic variants were identified in a total of 42 genes. Variants that explain the somatic phenotype but probably do not explain the DSD phenotype were observed in individuals (DSD cases 101 and 104), and one individual (DSD 93) carried two different variants that explain the DSD phenotype or somatic phenotype respectively. In the entire cohort 33.6% (42 individuals), carried either VUS/B/LB variants in 42 genes and in 16.8% (21 individuals) no variant could be linked to the phenotype. The diagnostic yield varied between the DSD subgroups. The subgroup initially diagnosed with 46, XY disorders of gonadal development, has 20% (3/15) individuals with P/LP variants and 60% (9/15) harbored VUS/LB/B variants. 46.3% (31/67) of patients diagnosed with “other DSD” harbored P/LP variants with 37.3% (25/67) carrying VUS/LB/B variants. The highest diagnostic yield was the syndromic 46, XY DSD subgroup where 69.2% (27/39) carried P/LP variants and 20.5% (8/39) carried VUS/LB/B variants. The overall diagnostic yield is similar to that reported recently in other large cohorts using a WES approach but higher than studies using an NGS gene panel (Baxter et al., 2015; Eggers et al., 2016; Kim et al., 2017; Ozen et al., 2017; Buonocore et al., 2019; Hughes et al., 2019; Xu et al., 2019; Abualsaud et al., 2020; Yu et al., 2021). Abualsaud et al. (2020) identified a genetic cause in 51% of a cohort of 149 DSD cases, using targeted DSD gene panel Yu et al. (2021) reported a diagnostic yield of 42.5% in 46, XY DSD whereas Xu et al. (2019) reported a diagnostic yield of 46.9% of 46, XY DSD and 10.3% of DSD 46, XX individuals. Selected samples without molecular causes were re-analysed by whole exome sequencing (WES) and the yield did not improve the diagnostic rate (Xu et al., 2019). Eggers et al. (2016), using a targeted DSD gene panel, identified a likely genetic diagnosis in 43% of patients in a large cohort of 46, XY DSD. Similarly, Baxter et al. (2015) in a cohort of 40 patients established a likely clinical genetic diagnosis of 35%. The increase in diagnostic yield in this study is due in part to the inclusion of new DSD genes that have been discovered by our group in the last decade and that were not included in the previous studies (e.g. *DHX37, ZFPM2*) and also the use of exome sequencing rather than the targeted gene panel. Targeted sequencing using DSD panels identify the genetic cause in approximately 30% of cases (Buonocore et al., 2019; Hughes et al., 2019).

All of these studies (and the current study) have important limitations and the diagnostic yields are imprecise. Variability in the diagnostic yield can be due to several factors including limited clinical data, unknown mode of inheritance of the variant as well as the absence of meaningful functional studies. An example of the latter are variants in the *GATA4* and *ZFPM2* genes which were classified as P/LP (Eggers et al., 2016). Subsequent functional studies resulted in the classification of most of these variants as VUS/LB/B (van den Bergen et al., 2020). A lower diagnostic yield can occur when pathogenic variants are difficult to identify for specific DSD genes. A good example of this are missense variants involving the protein kinase signal transduction factor MAP3K1. Variants in MAP3K1 are an established cause of 46, XY DSD (Pearlman et al., 2010). The vast majority of the published pathogenic variants are missense variants and limited functional data indicate that these missense variants may be gain-of-function rather than loss-of-function. Such missense variants are difficult to classify using *in silico* predictive tools and there is not a robust and simple functional assay to determine the consequences of MAP3K1 variants on biological function. This results in a tendency to classify MAP3K1 variants as VUS, when in fact a proportion of them are probably pathogenic (e.g. Eggers et al., 2016; Xu et al., 2019). The differences in diagnostic yield may also reflect differences in the constitution of the DSD cohort. The diagnostic yield of the undervirilised male, for example is much lower than that for 46, XY gonadal dysgenesis or disorders of androgen synthesis or action (Hughes et al., 2006). Other factors may influence the yield. Approximately 30% of the cases in this cohort are syndromic forms of XY DSD and the genetic etiology was identified in 69.2%of this cohort. Consanguinity may also contribute to the higher diagnostic yield with 36% of individuals reporting some form of parental consanguinity. This is reflected in the data with, for example, a total of nine individuals with a recessive form of DSD associated with biallelic variants in either *HSB17B3* or *SRD5A2*.

Eggers et al. (2016) reported variants in seven genes known to cause CHH, which were carried by 9% of patients with 46, XY DSD including primary hypogonadism, suggesting that these variants may contribute to a wider range of 46, XY DSD phenotypes. In 45 subjects, we identified43 variants involving the *ANOS1*, *CCDC141*, *CHD7, DUSP6, FEZF1, FGFR1, FLRT3, FSHB, GNRHR, HS6ST1, LHX3, LHX4, PLXNA3, PROK2, PROKR2, PROP1, RNF216, SEMA3A, SEME3F* and *SPRY4* genes*.* Within this group of genes, 17 P/LP variants were carried by 16.8% (21/125) of the cohort and these may contribute to the phenotype. Recently, hemizygous variants in the genes encoding the receptor for*SEMA3F* termed*PLXNA3* have been proposed as a cause of 46, XY CHH (Kotan et al., 2021). However, our data suggest that association of PLXNA3 LOF variants with CHH is unlikely, since one man (DSD 15), presented with gonadal dysgenesis/TRS and with elevated gonadotropins was found to harbor a hemizygous LOF variant in PLXNA3 (p.V263fs).

A comparative analysis of monozygotic twin boys discordant for DSD phenotype highlighted three novel variants of uncertain significance identified in the *CDCC141, SCLY* and *VEGFB* genes. The boys could either share variants that cause the phenotype but one boy is unaffected (incomplete penetrance) or the affected boy may carry a *de novo* pathogenic variant that arose during early fetal development. A small proportion of *de novo* variants in monogenic congenital disorders, that are discordant in monozygotic twin pairs have been reported [Kruyer et al., 1994; Taylor et al., 2008; Zwijnenburg et al., 2010]. Consistent with the hypothesis of incomplete penetrance, both brothers carried a maternally inherited heterozygous and novel frameshift variant (p.V1033fs) that is predicted to result in a truncated CCDC141 protein. Although both mono- and biallelic *CCDC141* variants are associated with CHH with or without anosmia (Turan et al., 2017), no evidence was found that would warrant a clinical diagnosis of CHH. Incomplete penetrance associated with truncating variants in CCDC141 have been described previously in a large Chinese cohort with CHH (Hou et al., 2020). We also identified two variants that were discordant between the affected and unaffected sibs. The first is a novel homozygous missense variant (p.P3L) in the *VEGFB* gene harbored by the proband and present in the heterozygous state in his unaffected brother. Both parents are heterozygous. VEGFB plays a key role in the regulation of blood vessel physiology, endothelial targeting of lipids to peripheral tissues and apoptotic cell death *via* the endothelial receptor VEGFR1 (Hagberg et al., 2010; Lal et al., 2018). The affected sib also carries a novel heterozygous and *de novo* missense variant in the *SCLY* gene that is not carried by his twin brother. The p.P2S amino acid change is absent from all public SNP databases. The *SCLY* gene encodes selenocysteine lyase, a pyridoxal 5′-phosphate-dependent enzyme that specifically catalyzes the decomposition of L-selenocysteine to L-alanine and elemental selenium (Mihara et al., 2000). In the mouse, the Sclyprotein is known to be expressed in Leydig cells and selenium acts directly on testosterone production (Seale et al., 2018). An independent DSD case (DSD 60) carried a heterozygous missense variant p.D154N in SCLY which is considered likely disease causing (REVEL score 0.579). This child presented with micropenis and bilateral cryptorchidism. The parents of this child were not available for study and the inheritance of the variant is unknown. These variants may contribute to the phenotype but without further supporting genetic or experimental evidence they are classified as VUS.

The chicken ovalbumin upstream promoter–transcription factor type II (COUP-TFII encoded by the gene *NR2F2*) is a member of the steroid/thyroid nuclear receptor superfamily, structurally related to the orphan nuclear receptor NR5A1 (Polvani et al., 2019). NR2F2 plays a major role in the mesenchymal-epithelial transition and in doing so is involved in the development of multiple organs and tissues by modulating the gene expression to promote cellular differentiation, proliferation, migration, survival, and intercellular communication (Polvani et al., 2019). *De novo* heterozygous *NR2F2* frameshift variants cause testicular development in 46, XX patients born with atypical male external genitalia, congenital heart disease (CHD) and other somatic features (Bashamboo et al., 2018; Carvalheira et al., 2019). In contrast, no pathogenic variants have been described to date in individuals with 46, XY DSD. In XY male mice NR2F2 is essential for the differentiation and function of fetal and adult Leydig cells (Kilcoyne et al., 2014). Inactivation of *Nr2f2* during prepubertal stages of male sexual development results in infertility, hypogonadism, and a block in spermatogenesis due to a failure of progenitor Leydig cells to mature (Qin et al., 2008). Here, we identified a novel *de novo*, likely disease causing (REVEL 0.992) variant within the ligand-binding domain of the *NR2F2* gene (p.R246H) in a 46, XY boy with micropenis and hypospadias. This suggests that variants NR2F2 may be responsible for 46, XY DSD although further genetic and experimental evidence to support this hypothesis are required.

In conclusion, this study shows the power of WES to identify genetic causes of DSD and the data suggest that di/oligogenic forms of DSD may be more common that previously supposed. The data also suggest that hemizygous variants in the semaphorin receptor PLXNA3 are not a cause of 46, XY CHH and that NR2F2 variants may contribute to 46, XY DSD.

## Data Availability

The data presented in the study are deposited in the ClinVar (https://www.ncbi.nlm.nih.gov/clinvar/) repository, accession numbers (VCV001202599.1, VCV000974911, VCV001202584, VCV001202585, VCV001202591.1, VCV001202603.1, VCV001202602.1, VCV000194727.30, VCV001199404.1, VCV001199394.3, VCV001202593.1, VCV001202605, VCV000800795.2, VCV001202594.1, VCV001202595.1, VCV001199404.1, VCV001199398.2, VCV001202601.1, VCV001202589, VCV001202586, VCV001199396.2, VCV001202600.1, VCV001202592.1, VCV001199405.1, VCV001199399.1, VCV001202590).
