# Supplementary material for: Novel Genomic Variants, Atypical Phenotypes and Evidence of a Digenic/Oligogenic Contribution to Disorders/Differences of Sex Development in a Large North African Cohort
Source: Front Genet. 2022 Aug 30;13:900574. doi: 10.3389/fgene.2022.900574 (PMC9468775; doi:10.3389/fgene.2022.900574)
Supplement: Supplementary file 1 [file Table1.docx]

**Table S1.** Clinical phenotype, hormonal profile and details of variants identified in individuals with 46 XY “other DSD”.

| **Case**  **Age**  **Sex of rearing** | **FSH (UI/l)** | **LH (UI/l)** | **Testosterone (ng/ml)** | | **AMH (ng/ml)** | **Genitourinary** | | | | **Variant** | **MAF and population (gnomAD)/ Predicted effect on protein** | **Zyg** | **PoGI/ transmission of variant** | **Clinicalsignificance: ACMG/ ClinVar/ GV, Ref** |
| --- | --- | --- | --- | --- | --- | --- | --- | --- | --- | --- | --- | --- | --- | --- |
|  | **Value/ NR** | **Value/ NR** | **Value/ NR** | **Value-HCG stimulation test/NR** | **Value/ NR** | **Externa genitalia** | **Internal genitalia** | **Gonadal position** | **Gonad/ Histology** |  |  |  |  |  |
| **17**  1 D  M | NA | 0.175 [0.80-7.60] (4Y) | 1.81 [0,12-4,04] (1Mo) | NA | 10.8 [15.5-48.5] (1Mo) | Phallus (2x1cm), developed scrotum, UC | No Müllerian ducts | R: scrotum, L: inguinal | R: testis/ NA, L: testis/ NA | GNRHR:NM_000406:c.C436T:p.P146S | 0.004587- Other/ SIFT: deleterious (0), PolyPhen2: probably damaging (1), REVEL: 0.656 (LDC) | Het | AR Maternal | VUS/ LB- VUS/ VUS [Stamou et al., 2019] |
|  |  |  |  |  |  |  |  |  |  | CCDC141:NM_173648:c.G2483A:p.R828Q | 0.0001202- African/African-American/ SIFT: tolerated (0.57), PolyPhen2: B (0.183), REVEL: 0.035 (B) | Het | AR Paternal | VUS/ NA/ NA |
| **18**  10 Mo  M | 1.02 [0,2-2,8] (4Y) | <0.1 [<0.2] (4Y) | <0.05 [<0.3] (16Mo) | NA | 70.3 [51.3-88.3] (16Mo) | Micropenis (2x0.8cm), posterior hypospadias, developed scrotum, UC | No Müllerian ducts, prostate present | R: inguinal, L: scrotum | R: testis/ NA, L: testis/ NA | SRD5A2:NM_000348.4:c.G537A:p.R179S | 0.00004860- European (non-Finnish)/ SIFT: deleterious (0), PolyPhen2: possibly damaging (0.754), REVEL: NA | Het | AR Maternal | VUS/ NA/ NA |
|  |  |  |  |  |  |  |  |  |  | GPRC6A:NM_148963:c.T1391A:p.F464Y | 0.01530- African/African-American/ SIFT: deleterious (0.01), PolyPhen2: probably damaging (1), REVEL: 0.740 (LDC) | Het | NA | B/ NA/ P [Jørgensen and Bräuner-Osborne, 2020] |
|  |  |  |  |  |  |  |  |  |  | SOS1:NM_005633:c.A244G:p.I82V | 0.0002780- Other/ SIFT: deleterious (0), PolyPhen2: probably damaging (0.974), REVEL: 0.416 (B) | Het | AD | VUS/ VUS/ NA |
| **19#**  2 Mo  F | 0.55 [0.2-11.1] (4Y) | <0.1 [0.1-0.5] (4Y) | <0.025 [0.12-0.21] (3Mo) | 7.850 [0.12-0.21] (10Mo) | 94.5 [42-203] (4Mo) | Clitoromegaly, perineal orifice, pigmented labioscrotal folds, BC, PG | No Müllerian ducts | R: inguinal, L: inguinal | R:hypotrophic testis/ NA, L: hypotrophic testis/ NA | SRD5A2:NM_000348.4:c.A704T:p.Y235F | 0.00002299- European (non-Finnish)/ SIFT: NA, PolyPhen2: NA, REVEL: NA | Hom | AR | P/ P/ P [Parlak et al., 2014] |
| **20 #**  4 Mo  M | NA | NA | 0.482 [0.12-0.32] (6Mo) | 2.430 [0.12-0.32] (8Mo) | >23 [16-140] (6Mo) | Micropenis (1cm), posterior hypospadias, hypoplastic pigmented labioscrotal folds, PG | No Müllerian ducts | R: inguinal, L: inguinal | R: testis/ NA, L: testis/ NA | HSD17B3:NM_000197:c.A679G:p.T227A | Novel/ SIFT: tolerated (0.71), PolyPhen2: B (0.04), REVEL: 0.567 (LDC) | Hom | AR | P/ NA/ NA |
|  |  |  |  |  |  |  |  |  |  | GPRC6A:NM_148963:c.C425T:p.A142V | Novel/ SIFT: tolerated (0.05), PolyPhen2: probably damaging (0.987), REVEL: 0.806 (LDC) | Het |  | VUS/ NA/ NA |
| **22**  2 Mo  M | NA | NA | NA | NA | 135.9 [16.8-193] (30Mo) | Curved micropenis, penoscrotal hypospadias, UC | No Müllerian ducts | R: scrotum, L: no residual gonad | R: testis/ NA, L: no residual testis | GHRHR:NM_000823:c.812+4A>C | Novel/ LOF | Het | AR | VUS/ NA/ NA |
|  |  |  |  |  |  |  |  |  |  | MYRF:NM_001127392:c.C848T:p.T283I | 0.0005191- European (non-Finnish)/ SIFT: tolerated (0.06), PolyPhen2: possibly damaging (0.801), REVEL: 0.105 (B) | Het | AD | VUS/ NA/ NA |
|  |  |  |  |  |  |  |  |  |  | PROP1:NM_006261:c.G302T:p.S101I | Novel/ SIFT: deleterious (0.03), PolyPhen2: B (0.419), REVEL: 0.311 (B) | Het | AR | VUS/ NA/ NA |
|  |  |  |  |  |  |  |  |  |  | FGFR2:NM_000141:c.G34A:p.V12M | 0.001362- African/African-American/ SIFT: tolerated low confidence (0.11), PolyPhen2: B (0.003), REVEL: 0.239 (B) | Het | AD | VUS/ LB/ VUS [Nakanishi et al., 2014] |
| **23 #**  2 D  M | 1.09 [<3] (2Mo) | 6.12 [<1] (2Mo) | 0.59 [0,42-0,8] (2Mo) | 2.89 [0,42-0,8] (18Mo) | 23.0 [16.0-140] (2Mo) | Micropenis (2cm), posterior hypospadias | No Müllerian ducts | R: scrotum, L: scrotum | R: testis/ NA, L: testis/ NA | FGFR1:NM_001174067:c.G211A:p.V71M | 0.0001255- African/African-American/ SIFT: tolerated (0.15), PolyPhen2: B (0.009), REVEL: 0.155 (B) | Het | AD Maternal | LP/ NA/ P [Wang et al., 2020] |
|  |  |  |  |  |  |  |  |  |  | HSD17B3:NM_000197:c.A679G:p.T227A | Novel/ SIFT: tolerated (0.71), PolyPhen2: B (0.04), REVEL: 0.567 (LDC) | Het | AR | VUS/ NA/ NA |
| **24 #**  4Y  M | NA | NA | 0.013 [0.03-0.38] (4Y) | NA | 23.0 [16.8-193] (4Y) | Micropenis (3.5cm), penoscrotal hypospadias, anogenital distance of 3 cm, developed scrotum, PG | No Müllerian ducts | R: scrotum, L: scortum | R: testis/ NA, L: oscillanting testis/ NA | MAMLD1:NM_001177465:c.C2573T:p.P858L | Novel/ SIFT: deleterious (0.03), PolyPhen2: possibly damaging (0.625), REVEL: 0.188 (B) | Hem | XLR | VUS/ NA/ NA |
|  |  |  |  |  |  |  |  |  |  | RXFP2:NM_130806:c.C1904T:p.A635V | Novel/ SIFT: deleterious (0.04), PolyPhen2: B (0.033), REVEL: 0.121 (B) | Het | AR | VUS/ NA/ NA |
|  |  |  |  |  |  |  |  |  |  | FLRT3:NM_013281:c.T325G:p.L109V | 0.00002897- Latino/ Admixed American/ SIFT: deleterious (0.02), PolyPhen2: probably damaging (0.98), REVEL: 0.738 (LDC) | Het | AD | LP/ NA/ NA |
|  |  |  |  |  |  |  |  |  |  | CTU2:NM_001012759:c.G1127A:p.R376Q | 0.01090- African/ African-American/ SIFT: tolerated (0.32), PolyPhen2: B (0.317), REVEL: 0.091 (B) | Het | AR | B/ B/ NA |
| **25 #**  2 D  M | NA | NA | <0.05 [0.05-0.3] (25Mo) | NA | >9,00 [36,4-194,6] (25Mo) | Micropenis (1.5cm), middle hypospadias, urethral orifice, urogenital sinus partially closed, BC | No Müllerian ducts | R: inguinal, L: inguinal | R: testis (17x6mm)/ NA, L: testis (14x5mm)/ NA | SEMA3A:NM_006080:c.G9C:p.W3C | 0.004770- African/ African-American/ SIFT: tolerated (0.15), PolyPhen2: tolerated (0), REVEL: 0.138 (B) | Het | AD | LP/ NA/ NA |
|  |  |  |  |  |  |  |  |  |  | CHD7:NM_017780:c.G6989C:p.G2330A | 0.002503- African/ African-American/ SIFT: deleterious (0), PolyPhen2: probably damaging (0.995), REVEL: 0.736 (LDC) | Het | AD | LP/ LB/ VUS [Bartels et al., 2010] |
| **26**  2 Y  M | <0.1 (2Y) | 0.67 (2Y) | <0.25 [1.75-7.81] (2Y) | 5.22 [1.75-7.81] (2Y) | 135.0 [42.0-185.0] (2Y) | Micropenis (2cm), penoscrotal hypospadias, urethral orifice, urogenital sinus closed, developed scrotum, PG | No Müllerian ducts | R: inguinal, L: inguinal | R: testis/ NA, L: testis/ NA | RNF216:NM_207111:c.G785A:p.R262H | 0.0005534- Other/ SIFT: tolerated low confidence (0.05), PolyPhen2: B (0.169), REVEL: 0.016 (B) | Het | AR | VUS/ NA/ NA |
|  |  |  |  |  |  |  |  |  |  | GLI2:NM_005270:c.G3528T:p.Q1176H | 0.005776- African/ African-American/ SIFT: deleterious (0), PolyPhen2: possibly damaging, REVEL: 0.171 (B) | Het | AD | VUS/ B/ VUS [Bodian et al., 2016] |
|  |  |  |  |  |  |  |  |  |  | MAMLD1:NM_001177465:c.C530T:p.T177M | 0.003219- European (Finnish)/ SIFT: deleterious (0), PolyPhen2: possibly damaging (0.481), REVEL: 0.156 (B) | Hem | XLR | VUS/ B/ VUS [Li et al.,2020] |
| **28**  3 Y  M | NA | NA | <0.05 [0.05-7] (3Y) | 0.99 [0.03-0.38] (4Y) | 21.41 [16.8-193] (3Y) | Micropenis (2.5cm), urethral orifice, urogenital sinus closed, BC | No Müllerian ducts | R: inguinal, L: inguinal | R: testis (10x6mm)/ NA, L: (12x5mm) testis/ NA | AMH:NM_000479:c.T991C:p.S331P | 0.002318- African/ African-American/ SIFT: deleterious (0), PolyPhen2: tolerated (0.322), REVEL: 0.730 (LDC) | Het | AR | VUS/ NA/ NA |
| **29 #**  10 Mo  M | NA | NA | 0.12 [<1.51] (12Mo) | NA | >9 [1.43-11.6] (12Mo) | Posterior hypospadias | No Müllerian ducts | R:scortum, L: scrotum | R: testis/ NA, L: testis/ NA | PROKR2:NM_144773:c.C253T:p.R85C | 0.002218- Ashkenazi Jewish/ SIFT: deleterious (0), PolyPhen2: probably damaging (1), REVEL: 0.422 (B) | Het | AD *De novo* | LP/ LP/ P [McCormack et al., 2017] |
|  |  |  |  |  |  |  |  |  |  | GLI2:NM_005270:c.2294-4G>A | 0.0009812- South Asian/ LOF | Het | AD | VUS/ B/ NA |
|  |  |  |  |  |  |  |  |  |  | GPRC6A:NM_148963:c.G1882A:p.G628R | 0.0001390- Other/ SIFT: deleterious (0), PolyPhen2: probaby damaging (0.998), REVEL: 0.776 (LDC) | Het |  | VUS/ NA/ NA |
| **30**  1 D  M | NA | NA | NA | NA | NA | Perineal hypospadias, urethral orifice, developed scrotum, UC, PG | No Müllerian ducts | R: scrotum, L: inguinal | R: testis/ NA, L: testis/ NA | ZNRF3:NM_001206998:c.G1507A:p.G503S | 0.0005872- Other/ SIFT: tolerated (0.13), PolyPhen2: B (0.097), REVEL: 0.144 (B) | Het |  | VUS/ VUS/ NA |
|  |  |  |  |  |  |  |  |  |  | GPRC6A:NM_148963:c.A1528C:p.N510H | 0.003367- African/African-American/ SIFT: tolerated (0.52), PolyPhen2: B (0.003), REVEL: 0.215 (B) | Het |  | VUS/ NA/ NA |
|  |  |  |  |  |  |  |  |  |  | POR:NM_000941:c.T976C:p.Y326H | Novel/ SIFT: deleterious (0.01), PolyPhen2: possibly damaging (0.79), REVEL: 0.703 (LDC) | Het | AR | VUS/ NA/ NA |
| **31**  4 Y  M | NA | NA | <0.05 [<0.2] (4Y) | 2.90 [0.03-0.32] (4Y) | 23.0 [3.8-159.8] (4Y) | Micropenis, posterior hypoaspadias, hypoplastic labioscrotal folds, BC | No Müllerian ducts | R: inguinal, L: inguinal | R: testis/ NA, L: testis/ NA | PLXNA3:NM_017514:c.C2170T:p.R724W | 0.00005319 African/African-American/ SIFT: deleterious (0), PolyPhen2: possibly damaging (0.586), REVEL: 0.084 (B) | Hem | AR | VUS/ NA/ NA |
| **32 #**  2 Y  M | 0.66 [3.7-10.4] (2Y) | <5 [0.2-0.4] (2Y) | <0.025 [0.01-0.1] (2Y) | NA | 23.0 [21-210] (2Y) | Micropenis, urethral orifice, developed scrotum, UC, PG | No Müllerian ducts | R: scrotum, L: inguinal | R: testis/ NA, L: testis/ NA | ZFPM2:NM_012082:c.A2501G:p.K834R | 0.006972- African/African-American/ SIFT: deleterious (0.01), PolyPhen2: probably damaging (0.985), REVEL: 0.718 (LDC) | Het | AD | LP/ B/ VUS [Longoni et al., 2015] |
| **34 #**  15 Y  F | 12.92 [3.35-21.63] (15Y) | 6.73 [1.14-8.7] (15Y) | 8.43 [0.1-0.9] (15Y) | NA | 2.9 [2.8-82] (15Y) | Micropenis (2-3cm), labia majora, urethral and vaginal orifice, NPG | Small uterus (49x12mm) present | No residual gonad | No residual gonad | SRD5A2:NM_000348.4:c.A704T:p.Y235F | 0.00002299- European (non-Finnish)/ SIFT: NA, PolyPhen2: NA, REVEL: NA | Hom | AR | P/ P/P [Parlak et al.,2014] |
|  |  |  |  |  |  |  |  |  |  | FANCD2:NM_033084:c.C2965G:p.P989A | 0.0004573- South Asian/ SIFT: deleterious (0.02), PolyPhen2: B (0.311), REVEL: 0.091 (B) | Het | AR | VUS/ VUS/ NA |
| **35 #**  10 D  M | NA | NA | NA | NA | NA | Perineal hypospadias | NA | NA | NA | HSD17B6:NM_003725:c.C830T:p.P277L | 0.001254- Ashkenazi Jewish/ SIFT: deleterious (0), PolyPhen2: probably damaging (0.997), REVEL: 0.744 (LDC) | Het | AR | VUS/ NA/ NA |
|  |  |  |  |  |  |  |  |  |  | FOXP1:NM_001244815:c.T16C:p.F6L | Novel/ SIFT: NA, PolyPhen2: B (0.159), REVEL score: 0.171 (B) | Het | AD | VUS/ NA/ NA |
|  |  |  |  |  |  |  |  |  |  | FGFR3:NM_001163213:c.G1885A:p.E629K | 0.0003111- Latino/Admixed American/ SIFT: deleterious (0), PolyPhen2: probably damaging (0.971), REVEL: 0.663 (LDC) | Het | AD | LP/ LB-VUS/ NA |
| **36 #**  26 Mo  M | NA | NA | 0.09 [0-0.10] (26Mo) | NA | 130 [16.8-193] (26Mo) | Micropenis, urogenital sinus closed, urethral orifice, developed scrotum, NPG | No Müllerian ducts | R: inguinal, L: inguinal | R: testis/ NA, L: testis/ NA | LHX4:NM_033343:c.G385A:p.E129K | 0.004807- African/African-American/ SIFT: deleterious (0.01), PolyPhen2: probably damaging (0.927), REVEL: 0.704 (LDC) | Het | AD | LP/ B/ NA |
|  |  |  |  |  |  |  |  |  |  | PLXNA3:NM_017514:c.C3944T:p.P1315L | 0.00007785 African/African American/ SIFT: deleterious (0), PolyPhen2: probably damaging (1), REVEL: 0.438 (B) | Hem | AR | VUS/ NA/ NA |
| **37**  23 Mo  M | 1.22 [2.0-12.0] (2Y) | 0.373 [1.50-12.40] (2Y) | <0.025 [2.25-9.0] (2Y) | 7.5 [2.25-9.0] (2Y) | NA | micropenis, middle hypospadias, urogenital sinus partially closed, urethral orifice, developed scrotum, PG | No Müllerian ducts | R: inguinal, L: inguinal | R: testis/ NA, L: testis/ NA | NR2F2:NM_021005:c.G737A:p.R246H | Novel/ SIFT: deleterious (0.03), PolyPhen2: probably damaging (0.988), REVEL: 0.922 (LDC) | Het | AD *De novo* | VUS/ NA/ NA |
|  |  |  |  |  |  |  |  |  |  | GLI2:NM_005270:c.C4661T:p.P1554L | 0.00002637- European (non-Finnish)/ SIFT: deleterious (0.03), PolyPhen2: possibly damaging (0.794) , REVEL: 0.381 (B) | Het | AD Paternal | VUS/ NA/ NA |
|  |  |  |  |  |  |  |  |  |  | GLI3:NM_000168:c.T3935G:p.M1312R | 0.001101- Latino/Admixed American/ SIFT: tolerated low confidence (0.06), PolyPhen2: B (0.0), REVEL: 0.068 (B) | Het | AD Paternal | VUS/ B-LB-VUS/ NA |
| **38 #**  10 Mo  M | NA | NA | 0.09 [9.9-27.8] (12Mo) | 0.80 [0,3-0,38] (29Mo) | 14.95 [3.8-159.8] (12Mo) | Micropenis, posterior hypospadias, urethral orifice, bifid labioscrotal folds, BC, NPLG | No Müllerian ducts | R: inguinal, L: inguinal | R: testis/ NA, L: testis/ NA | HSD17B3:NM_000197:c.A641G:p.E214G | 0.00002637- European (non-Finnish)/ SIFT: deleterious (0), PolyPhen2: B (0.012), REVEL: 0.250 (B) | Hom | AR | P/ VUS/ NA |
|  |  |  |  |  |  |  |  |  |  | ZNRF3:NM_001206998:c.G1507A:p.G503S | 0.0005872- Other/ SIFT: tolerated (0.14), PolyPhen2: B (0.097), REVEL: 0.144 (B) | Het |  | VUS/ VUS/ NA |
|  |  |  |  |  |  |  |  |  |  | ZFPM2:NM_012082:c.C2534T:p.T845M | 0.0002022- South Asian/ SIFT: tolerated (0.08), PolyPhen2: tolerated (0.127), REVEL: 0.144 (B) | Het | AD | VUS/ NA/ NA |
| **39**  12 Y  M | 1.04 [0.7-11.1] (12Y) | 0.759 [0.80-7.60] (12Y) | <0.025 [0.2-3] (12Y) | NA | 48.7 [4.2-105] (12Y) | Micropenis, urogenital sinus closed, urethral orifice, developed scrotum, BC, NPLG | NA | NA | NA | GNRHR:NM_000406:c.A317G:p.Q106R | 0.005983- Ashkenazi Jewish/ SIFT: deleterious (0), PolyPhen2: probably damaging (0.992), REVEL: 0.606 (LDC) | Het | AR | VUS/ P/ P [D’Argenio et al., 2021] |
|  |  |  |  |  |  |  |  |  |  | PLXNA3:NM_017514:c.G5570A:p.R1857Q | 0.0002381 European (non Finnish)/ SIFT: deleterious (0.03), PolyPhen2: possibly damaging (0.565), REVEL: 0.158 (B) | Hem | AR | VUS/ NA/ NA |
|  |  |  |  |  |  |  |  |  |  | BBS7:NM_176824:c.A1094T:p.Q365L | Novel/ SIFT: tolerated (0.45), PolyPhen2: B (0), REVEL: 0.256 (B) | Het | AR | VUS/ NA/ VUS [de Groota et al., 2017] |
|  |  |  |  |  |  |  |  |  |  | GHR:NM_001242399:c.A1007G:p.H336R | 0.00003525- European (non-Finnish)/ SIFT: tolerated (0.11), PolyPhen2: possibly damaging (0.493), REVEL: 0.335 (B) | Het | AD | VUS/ NA/ NA |
|  |  |  |  |  |  |  |  |  |  | AKR1C3:NM_001253908:c.A548T:p.K183M | 0.01133- African/African-American/ SIFT: deleterious (0), PolyPhen2: probably damaging (0.987), REVEL: 0.272 (B) | Het |  | LB/ NA/ VUS [Camats et al., 2018] |
| **40 #**  11.5 Y  M | NA | 0.53 (12Y) | 0.14 [3.0-10.6] (12Y) | NA | 136.94 [25.2-249.8] (12Y) | Micropenis, posterior hypospadias, urethral orifice, developed scrotum, PG | No Müllerian ducts | R: abdominal cavity, L: abdominal cavity | R: testis/ NA, L: testis/ NA | SRD5A2:NM_000348.4:c.G607A:p.G203S | 0.001003- East Asian/ SIFT: deleterious (0), PolyPhen2: probably damaging (0.999), REVEL: NA | Hom | AR | P/ P/ P [Yuan et al., 2017] |
|  |  |  |  |  |  |  |  |  |  | GHR:NM_001242399:c.G613A:p.E205K | Novel/ SIFT: deleterious (0.04), PolyPhen2: possibly damaging (0.571), REVEL: 0.461 (B) | Het | AD | VUS/ NA/ NA |
| **41 #**  7.5 Y  M | NA | NA | 0.025 [0.05-0.3] (7.5Y) | NA | 45.9 [12.6-167] (7.5Y) | Micropenis, urogenital sinus closed, developed scrotum, PG | No Müllerian ducts | R: scrotum, L: scrotum | R:oscillating testis/ NA, L: testis/ NA | CHD7:NM_017780:c.C8416G:p.L2806V | 0.004017- Latino/Admixed American/ SIFT: tolerated (0.31), PolyPhen2: possibly damaging (0.458), REVEL: 0.126 (B) | Het | AD | LP/ B- LB/ VUS [Alkelai et al.,2017] |
|  |  |  |  |  |  |  |  |  |  | TGIF1:NM_170695:c.T25C:p.S9P | 0.006396- African/African-American/ SIFT: NA, PolyPhen2: B (0), REVEL: 0.078 (B) | Het | AD | VUS/ VUS/ VUS VUS [Brauner et al., 2020] |
| **42**  8 Y  M | NA | NA | <0.025 [0.03-0.38] (8Y) | 0.27 [0.03-0.38] (8Y) | 40.5 [12.6-167] (8Y) | Micropenis, urethral orifice, developed scrotum, UC, PG | No Müllerianducts | R: scrotum, L: inguinal | R:hypotrophic testis/ NA, L: inguinal/ NA | FLRT3:NM_01328:c.C152T:p.S51F | Novel/ SIFT: tolerated (0.07), PolyPhen2: B (0.049), REVEL: 0.295 (B) | Het | AD Paternal | VUS/ NA/ NA |
|  |  |  |  |  |  |  |  |  |  | CYB5A:NM_001914:c.289-5G>C | Novel/ LOF | Het | AR | VUS/ NA/ NA |
|  |  |  |  |  |  |  |  |  |  | HSD17B6:NM_003725:c.G285T:p.Q95H | 0.0004687- Latino/Admixed American/ SIFT: deleterious (0.01), PolyPhen2: B (0.171), REVEL: 0.337 (B) | Het | AR | VUS/ NA/ NA |
|  |  |  |  |  |  |  |  |  |  | AKR1C4:NM_001818:c.C826T:p.R276W | Novel/ SIFT: deleterious (0.04), PolyPhen2: possibly damaging (0.651), REVEL: 0.218 (B) | Het | AR | VUS/ NA/ NA |
| **43 #**  31 Mo  F | NA | NA | <0.025 [0.03-0.36] (32Mo) | 6.59 [0.03-0.36] (34Mo) | 122.7 [16.8-193] (32Mo) | Female external genitalia, vaginal orifice, BC, NPG | Vaginal cavity (3cm) present | R: inguinal, L: inguinal | R: testis/ Testicular structure, L: testis/ Testicular testis | AR:NM_000044:c.A2236G:p.M746V | Novel/ SIFT: deleterious (0.01), PolyPhen2: probably damaging (0.917), REVEL: 0.939 (LDC) | Hem | XLR | P/NA/ NA |
| **44**  3 Y  M | NA | NA | NA | NA | 75.57 [25-219] (3Y) | Penis (5cm), , developed scrotum, BC, PG | No Müllerian ducts | R: inguinal, L: inguinal | R: testis/ NA, L: testis/ NA | DGKK:NM_001013742.4:c.G3353A:p.S1069N | 0.02865- Ashkenazi Jewish/ SIFT: tolerated (0.27), PolyPhen2: B (0.085), REVEL score: NA (B) | Hem | *De novo* | B/B/ NA |
| **45 #**  13 Mo  M | NA | NA | 0.061 [0.12-0.31] (13Mo) | 2.49 [0.03-0.38] (14M) | 23 [16.0-140] (13Mo) | Micropenis (2.5cm), middle hypospadias, urethral orifice, developed scrotum, PG | No Müllerian ducts | R: scrotum, L: scrotum | R: testis/ NA, L: testis/ NA | PROKR2:NM_144773:c.C868T:p.P290S | 0.0003668- Latino/Admixed American/ SIFT: deleterious (0), PolyPhen2: probably damaging (1), REVEL: 0.939 (LDC) | Het | AD Maternal | LP/ VUS/ VUS [Cox et al., 2018] |
|  |  |  |  |  |  |  |  |  |  | MYRF:NM_001127392:c.A1222G:p.I408V | 0.001779- Latino/Admixed American/ SIFT: tolerated (0.19), PolyPhen2: B (0.241), REVEL: 0.062 (B) | Het | AD Paternal | VUS/ NA/ NA |
|  |  |  |  |  |  |  |  |  |  | CHD7:NM_017780:c.C8416G:p.L2806V | 0.004017- Latino/Admixed American/ SIFT: tolerated (0.31), PolyPhen2: possibly damaging (0.458), REVEL: 0.126 (B) | Het | AD | LP/ B- LB/ VUS [Alkelai et al., 2017] |
| **46**  9.5 Y  M | 4.56 (9.5Y) | 0.163 (9.5Y) | <0.13 [1.56-8.77] (9.5Y) | <0.025 [0.1-0.9] (10Y) | 38.0 [4.2-105] (12Y) | Penis (4.5cm), penoscrotal hypospadias, urethral orifice, developed scrotum, BC, PG | No Müllerian ducts | R: abdominal cavity, L: abdominal cavity | R: testis/ NA, L: hypotrophic testis/ NA | GLI2:NM_005270:c.A445G:p.S149G | 0.00001470- European (non-Finnish)/ SIFT: deleterious (0.02), PolyPhen2: probably damaging (0.914), REVEL: 0.330 (B) | Het | AD Maternal | VUS/ NA/ NA |
|  |  |  |  |  |  |  |  |  |  | GLI3:NM_000168:c.G1393C:p.G465R | 0.007522- Ashkenazi Jewish/ SIFT: tolerated (0.07), PolyPhen2: tolerated (0.01), REVEL: 0.226 (B) | Het | AD | VUS/ B-LB​/ VUS [Ciceri et al., 2018] |
| **49 #**  4 Y  M | NA | NA | 0.4 | NA | 43.2 [16.8-193] (4Y) | Micropenis (3.4cm), urogenital sinus closed, urethral orifice, hypoplasic labioscrotal folds, PG | No Müllerian ducts | R: inguinal, L: inguinal | R: testis/ NA, L: testis/ NA | SHH:NM_000193:c.G1367C:p.G456A | Novel/ SIFT: tolerated (0.11), PolyPhen2: probably damaging (0.996), REVEL: 0.798 (LDC) | Het | AD *De novo* or paternal | P/ NA/ NA |
|  |  |  |  |  |  |  |  |  |  | GHR:NM_001242399:c.G427T:p.V143L | 0.0004005- African/ African-American/ SIFT: tolerated (0.67), PolyPhen2: probably damaging (0.946), REVEL: 0.224 (B) | Het | AD | VUS/ NA/ NA |
|  |  |  |  |  |  |  |  |  |  | LZTR1:NM_006767:c.C905T:p.A302V | 0.00009441- African/ African-American/ SIFT: deleterious (0.04), PolyPhen2: B (0.181), REVEL: 0.448 (B) | Het | AD/ AR | VUS/ VUS/ NA |
|  |  |  |  |  |  |  |  |  |  | FGFR3:NM_001163213:c.G1885A:p.E629K | 0.0003111- Latino/Admixed American/ SIFT: deleterious (0), PolyPhen2: probably damaging (0.971), REVEL: 0.663 (LDC) | Het | AD | LP/ LB-VUS/ NA |
|  |  |  |  |  |  |  |  |  |  | FLRT3:NM_013281:c.T88C:p.S30P | Novel/ SIFT: tolerated (0.19), PolyPhen2: B (0.001), REVEL: 0.101 (B) | Het | AD Maternal | VUS/ NA/ NA |
| **50 #**  2 D  F | 0.1 [1.5-12.4] (7D) | 0.132 [<0.05] (7D) | 1.7 [0.08-0.481] (2Mo) | 0,75 [0.03-0.38] (6Mo) | 38.3 [21-210] (6Mo) | Micropenis, posterior hypospadias, urethral orifice, asymmetric bifid and depigmented labioscrotal folds, PG, UC | No Müllerian ducts | R: inguinal, L: inguinal | R: testis/ NA, L: testis/ NA | HSD17B3:NM_000197:c.A139G:p.M47V | 0.00008516- European (non-Finnish)/ SIFT: deleterious (0.04), PolyPhen2: B (0.001), REVEL: 0.390 (B) | Hom | AR | P/ NA/ NA |
| **51**  2 D  M | 0.51 [<3] (5Mo) | 1.43 [<1] (5Mo) | 0.47 [0.025-0.383] (5Mo) | NA | 74.5 [16-140] (3Mo) | Micropenis (1.5cm), penoscrotal hypospadias, developed scrotum | No Müllerian ducts | R: inguinal, L: inguinal | R: testis/ NA, L: testis/ NA | ZNRF3:NM_001206998:c.G1861T:p.A621S | 0.00006541- Latino/Admixed American/ SIFT: tolerated low confidence (0.27), PolyPhen2: B (0.003), REVEL: 0.056 (B) | Het |  | VUS/ NA/ NA |
|  |  |  |  |  |  |  |  |  |  | DUSP6:NM_001946:c.C279G:p.D93E | 0.0001962- Latino/Admixed American/ SIFT: tolerated (0.92), PolyPhen2: B (0.058), REVEL: 0.206 (B) | Het | AD | VUS/ NA/ NA |
| **56**  3 D  M | NA | NA | NA | 4.474 [0.1-13] (20Mo) | 113 [38-180] (18Mo) | Micropenis, hypospadias, urethral orifice, developed scrotum, UC, NPG | No Müllerian ducts | R: inguinal, L: inguinal | R: testis/ NA, L: testis/ NA | PSMD12:PSMD12:NM_00281:c.T2C:p.M1T | Novel/ SIFT: deleterious low confidence (0), PolyPhen2: possibly damaging (0.599), REVEL: 0.482 (B) | Het | AD | VUS/ NA/ NA |
| **57 #**  19 Y  F | NA | NA | 6.5 [2.62-8.7] (19Y) | NA | NA | Micropenis, hypospadias, urethral orifice, hypoplastic scrotum, BC, NPG | No Müllerian ducts, curved short male urethra | R: no residual gonad, L: inguinal | R: no residual gonad, L: testis/ NA | SRD5A2:NM_000348.4:c.A704T:p.Y235F | 0.00002299- European (non-Finnish)/ SIFT: NA, PolyPhen2: NA, REVEL: NA | Hom | AR | P/ P/ P [Parlak et al., 2014] |
|  |  |  |  |  |  |  |  |  |  | PROKR2:NM_144773:c.C868T:p.P290S | 0.0003668- Latino/Admixed American/ SIFT: deleterious (0), PolyPhen2: probably damaging (1), REVEL: 0.939 (LDC) | Het | AD | LP/ VUS/ VUS [Cox et al., 2018] |
| **58**  2 D  M | NA | NA | 0.75 [2.62-8.7] (24Mo) | 4.2 [<0.2] (25Mo) | 37.36 [21-210] (24Mo) | Micropenis, penoscrotal hypospadias, urogenital sinus partially closed, developed scrotum, PG | No Müllerianducts | R: inguinal, L: inguinal | R: testis/ NA, L: testis/ NA | HSD17B3:NM_000197:c.A139G:p.M47V | 0.00008516- European (non-Finnish)/ SIFT: deleterious (0.04), PolyPhen2: B (0.001), REVEL: 0.390 (B) | Het | AR | VUS/ NA/ NA |
|  |  |  |  |  |  |  |  |  |  | RNF216:NM_207111:c.G422T:p.G141V | Novel/ SIFT: deleterious low confidence (0.01), PolyPhen2: probably damaging (0.997), REVEL: 0.151 (B) | Het | AR | VUS/ NA/ NA |
|  |  |  |  |  |  |  |  |  |  | GHR:NM_001242399:c.G427T:p.V143L | 0.0004005- African/ African-American/ SIFT: tolerated (0.67), PolyPhen2: probably damaging (0.946), REVEL: 0.224 (B) | Het | AD | VUS/ NA/ NA |
|  |  |  |  |  |  |  |  |  |  | GPRC6A:NM_148963:c.C1382G:p.S461X | Novel/ LOF | Het |  | VUS/ NA/ NA |
| **59**  22 Mo  M | NA | NA | 0.025 [0.03-0.32] (22Mo) | 3.58 [0.03-0.32] (3Y) | 23.0 [3.8-159.8] (22M) | Micropenis, penoscrotal hypospadias, urethral orifice, hypoplasic scrotum | No Müllerian ducts | R: scrotum, L: scrotum | R: testis/ NA, L: oscillanting testis/ NA | LZTR1:NM_006767:c.G2023A:p.G675R | 0.0001206- African/African-American/ SIFT: deleterious (0), PolyPhen2: probably damaging (0.997), REVEL: 0.738 (LDC) | Het | AD/ AR Maternal | VUS/ NA/ NA |
|  |  |  |  |  |  |  |  |  |  | FLNA:NM_001456:c.G6967A:p.D2323N | Novel/ SIFT: deleterious (0.02), PolyPhen2: tolerated (0.439), REVEL: 0.320 (B) | Hem | XLD | VUS/ NA/ NA |
| **60**  20 Mo  M | NA | NA | <0.025 [0.03-0.38] (21Mo) | 4.23 [0.03-0.38] (23Mo) | 44.8 [16-140] (21Mo) | Micropenis, urogenital sinus closed, urethral orifice, developed scrotum, BC, NPG | No Müllerian ducts | R: inguinal, L: inguinal | R: testis/ NA, L: testis/ NA | FANCD2:NM_033084:c.T311C:p.I104T | 0.004114- African/African-American/ SIFT: deleterious (0.03), PolyPhen2: B (0.135), REVEL: 0.085 (B) | Het | AR | VUS/ LB- VUS/ NA |
|  |  |  |  |  |  |  |  |  |  | FANCD2:NM_033084:c.C986G:p.A329G | 0.003696- African/African-American/ SIFT: tolerated (0.11), PolyPhen2: B (0.221), REVEL: 0.090 (B) | Het | AR | VUS/ LB/ NA |
|  |  |  |  |  |  |  |  |  |  | AKR1C4:NM_001818:c.C589T:p.L197F | 0.0000088106- European (non-Finnish)/ SIFT: tolerated (0.31), PolyPhen2: B (0.34), REVEL: 0.089 (B) | Het | AR | VUS/ NA/ NA |
|  |  |  |  |  |  |  |  |  |  | AKR1C3:NM_001253908:c.T36G:p.D12E | Novel/ SIFT: NA, PolyPhen2: NA, REVEL: NA | Het |  | VUS/ NA/ NA |
|  |  |  |  |  |  |  |  |  |  | RNF216:NM_207111:c.C1471A:p.Q491K | Novel/ SIFT: tolerated (0.06), PolyPhen2: possibly damaging (0.591), REVEL: 0.081 (B) | Het | AR | VUS/ NA/ NA |
| **61**  3 Y  M | 1.04 [<3] (3Y) | 0.20 [<1] (3Y) | <0.025 [0.03-0.38] (3Y) | 2.60 [0.03-0.38] (3Y) | 55.4 [16.8-193] (3Y) | Hypospadias, urethral orifice, hypoplastic scrotum, BC, NPG | No Müllerian ducts | R: inguinal, L: inguinal | R: testis/ NA, L: testis/ NA | POR:NM_000941:c.A538G:p.T180A | 0.0001323- European (non-Finnish)/ SIFT: deleterious (0.01), PolyPhen2: probably damaging (0.963), REVEL: 0.810 (LDC) | Het | AR | VUS/ NA/ NA |
| **62 #**  4 Y  M | NA | NA | NA | 4.16 [0.03-0.38] (4Y) | 23 [16.8-193] (4Y) | Penis (5cm), urogenital sinus partially closed, developed scrotum, NPG | No Müllerian ducts | R: inguinal, L: inguinal | R: testis/ NA, L: testis/ NA | CHD7:NM_017780:c.C8416G:p.L2806V | 0.004017- Latino/Admixed American/ SIFT: tolerated (0.31), PolyPhen2: possibly damaging (0.458), REVEL: 0.126 (B) | Het | AD | LP/ B- LB/ VUS [Alkelai et al.,2017] |
|  |  |  |  |  |  |  |  |  |  | PROKR2:NM_144773:c.C253T:p.R85C | 0.002218- Ashkenazi Jewish/ SIFT: deleterious (0), PolyPhen2: probably damaging (1), REVEL: 0.422 (B) | Het | AD | LP/ LP/ P [McCormack et al., 2017] |
|  |  |  |  |  |  |  |  |  |  | TGIF1:NM_170695:c.G195C:p.W65C | 0.001809- European (Finnish)/ SIFT: deleterious (0.01), PolyPhen2: possibly damaging (0.769), REVEL: 0.465 (B) | Het | AD | VUS/ NA/ NA |
|  |  |  |  |  |  |  |  |  |  | ANOS1:NM_000216:c.C1187T:p.S396L | 0.003935- Ashkenazi Jewish/ SIFT: deleterious (0), PolyPhen2: B (0.085), REVEL: 0.462 (B) | Hem | XLR | LP/ P/ LP [Dodé et al., 2006] |
| **63 #**  2 D  F | 0.5 (34D) | 0.3 (34D) | 2.3 [0.38-1.90] (34D) | NA | 133 [42-203] (3Mo) | Female external genitalia, anogenital distance of 2cm, PG | No Müllerian ducts | L:labia majora, R: labia majora | R: gonad/ NA, L: gonad/ NA | AR:NM_000044:c.C2521G:p.R841G | Novel/ SIFT: deleterious (0), PolyPhen2: probably damaging (0.999), REVEL: 0.913 (LDC) | Hem | AR | P/ NA/ NA |
|  |  |  |  |  |  |  |  |  |  | GHR:NM_001242399:c.G1516A:p.V506M | 0.0001651- Other/ SIFT: deleterious (0), PolyPhen2: possibly damaging (0.897), REVEL: 0.377 (B) | Het | AD | VUS/ NA/ NA |
| **64 #**  3 D  M | NA | NA | NA | NA | NA | Perineal hypospadias | NA | NA | NA | SRD5A2:NM_000348.4:c.A704T:p.Y235F | 0.00002299- European (non-Finnish)/ SIFT: NA, PolyPhen2: NA, REVEL: NA | Het | AR | VUS/ P/ P [Parlak et al., 2014] |
|  |  |  |  |  |  |  |  |  |  | ZFPM2:NM_012082:c.G3055T:p.A1019S | 0.00003543- European (non-Finnish)/ SIFT: tolerated low confidence (0.07), PolyPhen2: B (0.039), REVEL: 0.042 (B) | Het | AD | LP/ NA/ NA |
| **65**  2 D  M | NA | NA | 0.05 [<0.2] (14Mo) | 3.45 [0.03-0.38] (12Mo) | 47.9 [21-210] (12Mo) | Hypoplastic pigmented scrotum, NPG | No Müllerian ducts | R: inguinal, L: inguinal | R:hypotrophic testis/ NA , L: hypotrophic testis/ NA | FGFR2:NM_022970:c.C1067G:p.T356S | 0.0003301- Other/ SIFT: tolerated (0.07), PolyPhen2: B (0.028), REVEL: 0.270 (B) | Het | AD | VUS/ NA/ NA |
| **66 #**  NA  M | NA | NA | NA | NA | NA | Micropenis, perineal hypospadias, UC | NA | NA | NA | RXFP2:NM_130806:c.G184A:p.A62T | 0.001524- Other/ SIFT: tolerated (0.43), PolyPhen2: B (0.035), REVEL: 0.281 (B) | Het | AR | VUS/ NA/ NA |
|  |  |  |  |  |  |  |  |  |  | PROKR2:NM_144773:c.C253T:p.R85C | 0.002218- Ashkenazi Jewish/ SIFT: deleterious (0), PolyPhen2: probably damaging (1), REVEL: 0.422 (B) | Het | AD | LP/ LP/ P [McCormack et al., 2017] |
|  |  |  |  |  |  |  |  |  |  | MAMLD1:NM_001177465:c.T1804C:p.S602P | Novel/ SIFT: tolerated low confidence (0.23), PolyPhen2: B (0), REVEL: 0.178 (B) | Hem | XLR | VUS/ NA/ NA |
|  |  |  |  |  |  |  |  |  |  | DCAF17:NM_025000:c.1422+3G>A | 0.0001161- Latino/Admixed American/ LOF | Het | AR | VUS/ NA/ NA |
| **67 #**  7 Y  M | 6.16 [1.5-12.4] (37Y) | NA | 11.20 [2.8-8.0] (37Y) | NA | NA | Micropenis, perineal hypospadias, UC | NA | R: no residual gonad, L: scrotum | R: no residual gonad, L: testis/ NA | RXFP2:NM_130806:c.G184A:p.A62T | 0.001524- Other/ SIFT: tolerated (0.43), PolyPhen2: B (0.035), REVEL: 0.281 (B) | Het | AR | VUS/ NA/ NA |
|  |  |  |  |  |  |  |  |  |  | PROKR2:NM_144773:c.C253T:p.R85C | 0.002218- Ashkenazi Jewish/ SIFT: deleterious (0), PolyPhen2: probably damaging (1), REVEL: 0.422 (B) | Het | AD | LP/ LP/ P [McCormack et al., 2017] |
|  |  |  |  |  |  |  |  |  |  | MAMLD1:NM_001177465:c.T1804C:p.S602P | Novel/ SIFT: tolerated low confidence (0.23), PolyPhen2: B (0), REVEL: 0.178 (B) | Hem | XLR | VUS/ NA/ NA |
| **68 #**  2 D  M | NA | NA | 1.81 [<10.96] (33Mo) | 3.7 [3.0-12.0] (33.5Mo) | 55.6 [51.3-88.3] (33M) | Micropenis (2.8cm), hypospadias, NPLG | No Müllerian ducts | R: scrotum, L: inguinal | R: testis/ NA, L: testis/ NA | SPRY4:NM_030964:c.G46A:p.V16I | 0.0006351- Latino/Admixed American/ SIFT: tolerated low confidence (0.19), PolyPhen2: B (0.015), REVEL: 0.027 (B) | Het | AD Maternal | LP/ NA/ LB [Stamou et al., 2019] |
| **69**  19 D  M | NA | NA | 0.0087 [3.0-12.0] (21Mo) | 3.95 | 73.7 [51.3-88.3] (24Mo) | Micropenis, perineal hypospadias, asymmetric labioscrotal folds, BC, NPG | Müllerian ductspresent | R: inguinal, L: inguinal | R: hypotrophic testis/ NA, L: testis/ NA | BBS12:NM_001178007:c.T1658C:p.L553P | Novel/ SIFT: deleterious (0), PolyPhen2: possibly damaging (0.876), REVEL: 0.605 (LDC) | Het | AR | VUS/ NA/ NA |
| **71**  5 D  M | NA | NA | 2.35 [<0.007] (16D) | NA | 40.3 [39.1-91.1] (2.5Mo) | Curved micropenis (3cm), penoscrotal hypospadias, developed scrotum, NPLG | No Müllerian ducts | R: inguinal, L: inguinal | R: hypotrophic testis/ NA, L: hypotrophic testis/ NA | SCLY:NM_016510:c.C4T:p.P2S | Novel/ SIFT: deleterious low confidence (0), PolyPhen2: B (0), REVEL: 0.005 (B) | Het | *De novo* | VUS/ NA/ NA |
|  |  |  |  |  |  |  |  |  |  | VEGFB:NM_001243733:c.C8T:p.P3L | Novel/ SIFT: deleterious low confidence (0.02), PolyPhen2: B (0.072), REVEL: 0.171 (B) | Hom |  | VUS/ NA/ NA |
|  |  |  |  |  |  |  |  |  |  | CCDC141:NM_173648:c.3098dupT:p.V1033fs | Novel/ NA | Het | AR Maternal | VUS/ NA/ NA |
| **72 #**  18 D  M | NA | NA | NA | NA | 18.14 [16.8-138] (20D) | Genital bud ,penoscrotal hypospadias, poorly developed scrotum, NPG | No Müllerian ducts | R: inguinal, L: inguinal | R: testis/ NA, L: testis/ NA | NR5A1:NM_004959:c.G206T:p.R69L | Novel/ SIFT: deleterious (0.01), PolyPhen2: probably damaging (1), REVEL: 0.957 (LDC) | Het | AD *De novo* | P/ NA/ NA |
|  |  |  |  |  |  |  |  |  |  | FLRT3:NM_013281:c.G950A:p.R317H | 0.002083- African/ African-American/ SIFT: tolerated (0.58), PolyPhen2: B (0.019), REVEL: 0.218 (B) | Het | AD | VUS/ NA/ NA |
| **73 #**  29 D  M | NA | NA | NA | NA | 78.80 [16.8-138] (1Mo) | Genital bud, preputial orifice and perineal orifice, bifid labioscrotal folds, BC, NPG | No Müllerianducts | R: inguinal, L: inguinal | R: testis/ NA, L: testis/ NA | MAMLD1:NM_005491:c.C2075T:p.A692V | Novel/ SIFT: deleterious low confidence (0), PolyPhen2: probably damaging (0.99), REVEL: 0.138 (B) | Hem | XLR | VUS/ NA/ NA |
|  |  |  |  |  |  |  |  |  |  | DMRT1:NM_021951:c.G149C:p.S50T | Novel/ SIFT: tolerated low confidence (0.45), PolyPhen2: B (0.122), REVEL: 0.069 (B) | Het | AD *De novo* | LP/ NA/ NA |
| **74 #**  3 Y  F | 3.62 (4.5Y) | 0.44 (4.5Y) | 0.02 [0.05-0.73] (3Y) | 0.07 (3.5Y) | >196 [<7] (3Y) | Femal external genitalia, clitoromegaly (2cm), urethral orifice | No Müllerian ducts | R: inguinal, L: inguinal | R + L testis/ atrophic seminiferous tubes with Sertoli cells, no germ cells, rare Leydig cells | LHCG:NM_000233:c.536+1G>A | Novel/ LOF | Hom | AR | P/ NA/ NA |
|  |  |  |  |  |  |  |  |  |  | SHH:NM_001310462:c.C423A:p.C141X | Novel/ LOF | Het | AD | P/ NA/ NA |
| **75 #**  2 D  M | NA | NA | 0.71 [1.75-7.81] (4Mo) | NA | <62.0 [21-210] (6.5Mo) | Buried micropenis (10x6mm), perineal hypospadias, urethral orifice, bifid scrotum, PG | No Müllerian ducts | R: scrotum, L: scrotum | R: testis (10x6mm)/ NA, L: testis (8x7mm)/ NA | NR0B1:NM_000475:c.C16A:p.H6N | 0.003316- Ashkenazi Jewish/ SIFT: tolerated low confidence (0.05), PolyPhen2: B (0.023), REVEL: 0.304 (B) | Hem | XL | VUS/ B- LB/ NA |
|  |  |  |  |  |  |  |  |  |  | CHD7:NM_017780:c.C1696G:p.P566A | 0.00003536- Latino/Admixed American/ SIFT: deleterious low confidence (0.05), PolyPhen2: B (0), REVEL: 0.088 (B) | Het | AD Paternal | LP/ VUS/ VUS[Brauner et al., 2020] |
| **76**  10 Mo  M | NA | NA | <0.025 [0.03-0.38] (12Mo) | NA | 128.7 [21-210] (12Mo) | Micropenis (2.5cm), posterior hypospadias, urogenital sinus partially closed, urethral orifice, developed scrotum, BC, NPRG | No Müllerian ducts | R: inguinal, L: inguinal | R: testis/ NA, L: testis/ NA | GLI2:NM_005270:c.A1811C:p.H604P | 0.0005540- Other/ SIFT: tolerated (0.38), PolyPhen2: B (0.009), REVEL: 0.178 (B) | Het | AD | VUS/ NA/ NA |
|  |  |  |  |  |  |  |  |  |  | HSD17B6:NM_003725:c.G285T:p.Q95H | 0.0004687- Latino/Admixed American/ SIFT: deleterious (0.01), PolyPhen2: B (0.171), REVEL: 0.337 (B) | Het | AR | VUS/ NA/ NA |
|  |  |  |  |  |  |  |  |  |  | FOXP1:NM_001244813:c.G1501A:p.A501T | 0.0002506- East Asian/ SIFT: 0.04 (deleterious), PolyPhen2: B (0), REVEL: 0.332 (B) | Het | AD | VUS/ NA/ NA |
|  |  |  |  |  |  |  |  |  |  | HS6ST1:NM_004807:c.C1144T:p.R382W | 0.01103- South Asian/ SIFT: deleterious (0), PolyPhen2: possibly damaging (0.73), REVEL: 0.634 (LDC) | Het | AD | LB/ B- LP- VUS/ VUS [Cangiano et al., 2019] |
| **77 #**  30 Mo  M | NA | NA | 0.07 [<0.2] (30Mo) | NA | 42.6 [16.8-193] (30Mo) | Curved micropenis, posterior hypospadias, urogenital sinus partially closed, urethral orifice, labia majora, BC | No Müllerian ducts | R: inguinal, L: inguinal | R: testis (13,05x06x11)/ NA, L: testis (13x06x10mm)/ NA | CCDC141:NM_173648:c.3474+5A>G | Novel/ NA | Het | AR | VUS/ NA/ NA |
|  |  |  |  |  |  |  |  |  |  | GHR:NM_001242399:c.T1563G:p.C521W | 0.003565- African/ African-American/ SIFT: tolerated (0.19), PolyPhen2: B (0.03), REVEL: 0.202 (B) | Het | AD | LP/ LB/ NA |
| **78 #**  29 Mo  M | NA | NA | 0.13 [2.2-10.5] (29Mo) | 5.7 | 139.8 [16.8-193] (3Y) | Micropenis, penoscrotal hypospadias, urethral orifice, developed scrotum, PG | No Müllerian ducts | R:scortum, L: scrotum | R: testis/ NA, L: testis/ NA | HSD17B3:NM_000197:c.C476A:p.T159N | Novel/ SIFT: tolerated (0.15), Polyphen2: B (0.038), REVEL: 0.506 (LDC) | Hom | AR | P/ NA/ NA |
| **79**  30 Mo  M | 1.64 [0.70-11.1] (30Mo) | 0.295 [0.80-7.60] (30Mo) | <0.3 [0.03-0.38] (30Mo) | 5.13 [0.03-0.38] (31Mo) | >45.9 [43-199] (30Mo) | micropenis, urethral orifice, hypoplastic pigmented scrotum, NPG | No Müllerian ducts | No residual gonad | NA | CTU2:NM_001012759:c.C710T:p.A237V | 0.0009344- European (Finnish)/ SIFT: deleterious (0), PolyPhen2: possibly damaging (0.581), REVEL: 0.184 (B) | Het | AD | VUS/ NA/ NA |
| **81 #**  16 Y  F | 0.86 [1.8-12] | 10.0 [0.5-12] | 9.57 [2.25-9] (16Y) | 16.48 | NA | Micropenis, penoscrotal hypospadias, bifid hypoplastic labioscrotal folds, NPG | No Müllerian ducts | R: inguinal, L: inguinal | R: testis/ NA, L: testis/ NA | SPRY4:NM_030964:c.T29A:p.L10Q | Novel/ SIFT: deleterious low confidence (0.01), PolyPhen2: probably damaging (0.998), REVEL: 0.108 (B) | Het | AD | LP/ NA/ NA |
|  |  |  |  |  |  |  |  |  |  | DCAF17:NM_025000:c.T1547C:p.I516T | 0.00001470- European (non-Finnish)/ SIFT: tolerated low confidence (0.42), PolyPhen2: B (0.0), REVEL: 0.070 (B) | Het | AR | VUS/ NA/ NA |
| **82**  20 Y  F | NA | NA | NA | NA | NA | Peniform organ and fusion of the labia minora | NA | NA | NA | SOS1:NM_005633:c.3347-1G>A | 0.0002259- European (non-Finnish)/LOF | Het | AD Maternal | VUS /VUS/ NA |
| **83**  8 Y  M | NA | NA | NA | NA | NA | Posterior hypospadias | NA | NA | NA | FGFR2:NM_000141:c.A1132G:p.I378V | Novel/ SIFT: tolerated (0.37), PolyPhen2: possibly damaging (0.503), REVEL: 0.290 (B) | Het | AD | VUS/ NA/ NA |
|  |  |  |  |  |  |  |  |  |  | FANCD2:NM_033084:c.C2965G:p.P989A | 0.0004573- South Asian/ SIFT: deleterious (0.02), PolyPhen2: B (0.311), REVEL: 0.091 (B) | Het | AR | VUS/ VUS/ NA |

**ACMG** American College of Medical Genetics, **AD** autosomal dominant, **AMH** anti-Müllerian hormone, **AR** autosomal recessive, **B** benign, **BC** bilateral cryptorchidism, **FSH** follicle stimulating hormone, **gnomAD** genome aggregation database, **GV** gene variants previously associated with the disease, **Het** heterozygous, **HCG** human chorionic gonadotropins, **Hom** homozygous, **L** left, **LB** likely benign, **LH** luteinizing hormone, **LOF** loss-of-function, **M** male, **MAF** minor allele frequency, **Mo** month, **NA** not available, **NR** normal range, **P** pathogenic, **PMDS** persistent Müllerian duct syndrome, **PoGI** Pattern of disease inheritance usually associated with the gene, **R** right, **LB** likely benign, **Ref** reference, **REVEL** rare exome variant ensemble learner. **^#^**A definitive genetic diagnosis was achieved
