# Supplementary material for: Novel Genomic Variants, Atypical Phenotypes and Evidence of a Digenic/Oligogenic Contribution to Disorders/Differences of Sex Development in a Large North African Cohort
Source: Front Genet. 2022 Aug 30;13:900574. doi: 10.3389/fgene.2022.900574 (PMC9468775; doi:10.3389/fgene.2022.900574)
Supplement: Supplementary file 2 [file Table2.docx]

**Table S2.** Clinical phenotype, hormonal profile and details of variants identified in individuals with syndromic 46,XY DSD.

| **Case**  **Age**  **Sex of rearing** | **FSH (UI/l)** | **LH (UI/l)** | **Testosterone (ng/ml)** | | **AMH (ng/ml)** | **Genitourinary** | | | | **Somatic features** | **Variant** | **MAF and population (gnomAD)/ Predicted effect on protein** | **Zyg** | **PoGI/ Transmission of variant** | **Clinical significance: ACMG/ ClinVar/GV, Ref** |
| --- | --- | --- | --- | --- | --- | --- | --- | --- | --- | --- | --- | --- | --- | --- | --- |
|  | **Value/ NR** | **Value/ NR** | **Value/ NR** | **Value-HCG stimulation test/ NR** | **Value/ NR** | **External genitalia** | **Internal genitalia** | **Gonadal position** | **Gonad/ histology** |  |  |  |  |  |  |
| **84**  1 D  M | NA | NA | NA | NA | NA | Micropenis, penoscrotal hypospadias, depigmented hypoplastic labioscrotal folds, NPG | NA | NA | NA | Polymalformative syndrome | KAT6B:NM_001256468:c.A1946T:p.N649I | 0.0005926- Latino/ Admixed American/ SIFT: deleterious (0), PolyPhen2: probably damaging, REVEL: 0.600 (LDC) | Het | AD | VUS/ NA/ NA |
| **86 #**  12 D  F | 2.23 [1,5-12,4] (2M) | 0.715 [<0.5] (2M) | 0.02 [2.49-8.36] (2M) | NA | 8.83 [42-203] (4M) | Micropenis, posterior hypospadias, pigmented hypoplastic genital labia, BC, NPG | No Müllerian ducts | R: inguinal, L: inguinal | R: testis (10.6x6.3mm)/ NA, L: testis (11.4x6.5mm)/ NA | Mute left kidney | NR5A1:NM_004959:c.T904C:p.W302R | Novel/ SIFT: deleterious (0), PolyPhen2: probably damaging (1), REVEL: 0.979 (LDC) | Het | AD *De novo* | P/ NA/ NA |
|  |  |  |  |  |  |  |  |  |  |  | HSD17B6:NM_003725:c.C830T:p.P277L | 0.001254- Ashkenazi Jewish/ SIFT: deleterious (0), PolyPhen2: probably damaging (0.997), REVEL: 0.744 (LDC) | Het | AR | VUS/ NA/ NA |
|  |  |  |  |  |  |  |  |  |  |  | PROK2:NM_001126128:c.96+4A>G | Novel/ LOF | Het | AD | LP/ NA/ NA |
|  |  |  |  |  |  |  |  |  |  |  | SEMA3F:NM_004186:c.A1480G:p.I494V | 0.00002896- Latino/ Admixed American/ SIFT: tolerated (0.72), PolyPhen2: B (0.159), REVEL: 0.170 (B) | Hem | AD | VUS/ NA/ NA |
| **87 #**  21 D  M | 0.965 [1.5-12.4] (45D) | 0.1 [<0.5] (45D) | 0.973 [2.49-8.36] (45D) | NA | 24.21 [25-175] (45D) | Micropenis, urethral orifice, pigmented and developed scrotum, BC, NPG | No Müllerian ducts | No residual gonad | No residual gonad | Cleft lip, cleft palate, lingual frenulum, ptosis, postaxial polydactyly, right ventricular chamber dilation, pulmonary hypertension, right renal nephrocalcinosis, mild left hydronephrosis | NR5A1:NM_004959:c.C1052T:p.A351V | 0.00009957- European (non-Finnish)/ SIFT: deleterious (0.02), PolyPhen2: possibly damaging (0.545), REVEL score: 0.727 (LDC) | Het | AD Maternal | P/ NA/ P [Cannarella et al., 2020] |
|  |  |  |  |  |  |  |  |  |  |  | PROKR2:NM_144773:c.C253T:p.R85C | 0.002218- Ashkenazi Jewish/ SIFT: deleterious (0), PolyPhen2: probably damaging (1), REVEL: 0.422 (B) | Het | AD | LP/ LP/ P [McCormack et al., 2017] |
|  |  |  |  |  |  |  |  |  |  |  | FGFR3:NM_001163213:c.C1498T:p.R500W | 0.0003190- African/ African-American/ SIFT: deleterious (0.01), PolyPhen2: probably damaging (0.952), REVEL: 0.595 (LDC) | Het | AD | LP/ NA/ NA |
|  |  |  |  |  |  |  |  |  |  |  | GLI2:NM_005270 :c.G3528T:p.Q1176H | 0.005776- African/ African-American/ SIFT: deleterious (0), PolyPhen2: possibly damaging, REVEL: 0.171 (B) | Het | AD | VUS/ B/ VUS [Bodian et al., 2016] |
|  |  |  |  |  |  |  |  |  |  |  | GPRC6A:NM_148963:c.A1528C:p.N510H | 0.003367- African/African-American/ SIFT: tolerated (0.52), PolyPhen2: B (0.003), REVEL: 0.215 (B) | Het |  | VUS/ NA/ NA |
|  |  |  |  |  |  |  |  |  |  |  | FLNA:NM_001110556:c.5218-4G>A | Novel/ LOF | Hem | XLD | P/ NA/ P [Alharbi et al., 2018] |
| **89 #**  1 D  M | NA | NA | <0.07 [<0.2] (23Mo) | 0.07 [<0.2] (3Y) | 4.24 [21-210] (2Y) | Micropenis (1cm), urethral orifice, urogenital sinus closed, hypoplastic depigmented scrotum, BC | No Müllerian ducts | R: inguinal, L: inguinal | R:hypotrophic testis/ NA, L: hypotrophic testis/ NA | Cleft lip, cleft palate, ptosis, posteriorly rotated ears, oligodactyly, syndactyly, psychomotor retardation, delayed fontanel closure | FGFR1:NM_001174067:c.G1960T:p.D654Y | Novel/ SIFT: deleterious (0), PolyPhen2: probably damaging (1), REVEL: 0.974 (LDC) | Het | AD | LP/ NA/ P [Simonis et al., 2013] |
|  |  |  |  |  |  |  |  |  |  |  | GHR:NM_001242399:c.G427T:p.V143L | 0.0004005- African/ African-American/ SIFT: tolerated (0.67), PolyPhen2: probably damaging (0.946), REVEL: 0.224 (B) | Het | AD Paternal | VUS/ NA/ NA |
|  |  |  |  |  |  |  |  |  |  |  | GHR:NM_001242399:c.C1449G:p.S483R | Novel/ SIFT: deleterious (0.04), PolyPhen2: possibly damaging (0.889), REVEL: 0.229 (B) | Het | AD Maternal | VUS/ NA/ NA |
| **90**  20 Mo  M | NA | NA | <0.025 [2.27-10.30] (17Mo) | 6.32 [2.27-10.30] (20Mo) | <9 [21-210] (17Mo) | Curved micropenis (2.5cm), anterior hypospadias, urethral orifice, hyposplastic pigmented labioscrotal folds, BC, NPG | No Müllerian ducts | R: inguinal, L: inguinal | R: testis/ NA, L: testis/ NA | Hirsutism, exotropia, short neck, arthrogryposis, kyphoscoliosis, hernia, axonal degeneration of the 4 limbs, peripheral motor neuropathy pure | AMH:NM_000479:c.T991C:p.S331P | 0.002318- African/African-American/ SIFT: deleterious (0), PolyPhen2: B (0.322), REVEL: 0.730 (LDC) | Het | AR | VUS/ NA/ NA |
|  |  |  |  |  |  |  |  |  |  |  | SLC29A3:NM_018344:c.G1088A:p.R363Q | 0.00002891- Latino/ Admixed American/ SIFT: deleterious (0), PolyPhen2: probably damaging (0.999), REVEL: 0.741 (LDC) | Het | AR Maternal | VUS/ P/ P [Capalbo et al., 2019] |
| **91 #**  3 Y  M | NA | NA | NA | NA | NA | Penoscrotal hypospadias, developed scrotum, PG | No Müllerian ducts | R:scortum, L: scrotum | R: testis/ NA, L: testis/ NA | Mental retardation, height and weight growth delays, opsoclonus-myoclonus syndrome | ZFPM2:NM_012082:c.A74G:p.E25G | 0.0002481- African/African-American/ SIFT: deleterious (0.02), PolyPhen2: B (0.123), REVEL: 0.154 (B) | Het | AD | LP/ NA/ NA |
| **92 #**  4 Y  M | NA | NA | NA | 1.28 [0.03-0.68] (5Y) | 23.0 [3.8-159.8] (4Y) | Phallus (3cm), urogenital sinus closed, urethral orifice, hypoplastic pigmented labioscrotal folds, BC, PG | No Müllerianducts | R: inguinal, L: inguinal | R:hypotrophic testis/ NA, L: hypotrophic testis/ NA | Mental retardation, height and weight growth delay, protruding ears, delayed tooth eruption | KAT6B:NM_001256468:c.A4822G:p.N1608D | Novel/ SIFT: deleterious low confidence (0.01), PolyPhen2: probably damaging (0.975), REVEL: 0.397 (B) | Het | AD *De novo* or paternal | P/ NA/ NA |
|  |  |  |  |  |  |  |  |  |  |  | GPRC6A:NM_148963:c.2323dupT:p.Y775fs | 0.2768- African/African-American/ LOF | Het | Maternal | B/ B/ VUS [Dawson et al., 2020] |
| **93 #**  4.5 Y  M | 0.7 [0.95-11.95] (5Y) | 0.15 [1.14-8.75] (5Y) | NA | 1,73 [1,75-71,81] (5Y) | 78.0 [3.8-159.8] (5Y) | Micropenis (2.5cm), penoscrotal hypospadias, urethral orifice, urogenital sinus closed, hypoplastic lablioscrotal folds, BC | No Müllerian ducts | R: inguinal, L: inguinal | R: testis/ NA, L: testis/ NA | Severe height and weight growth delay. Facial dysmorphia, short limb dwarfism, chest wall deformity, congenital hip dysplasia type III, abdominal bloating, pituitary microadenoma, Iron deficiency anemia | HSD17B3:NM_000197:c.C476A:p.T159N | Novel/ SIFT: tolerated (0.15), Polyphen2: B (0.038), REVEL: 0.506 (LDC) | Hom | AR | P/ NA/ NA |
|  |  |  |  |  |  |  |  |  |  |  | COL1A1:NM_000088:c.C739T:p.P247S | Novel/ SIFT: tolerated (0.17), PolyPhen2: B (0.003), REVEL: 0.439 (B) | Het | AD | LP/ NA/ NA |
|  |  |  |  |  |  |  |  |  |  |  | ATP7B:NM_000053:1:c.C4301T:p.T1434M | 0.008979- Ashkenazi Jewish/ SIFT: tolerated (0.1), PolyPhen2: possibly damaging (0.454), REVEL: 0.199 (B) | Hom | AD | VUS/ B- LB- VUS/ VUS [Collet et al.,2018] |
|  |  |  |  |  |  |  |  |  |  |  | GPC3:NM_001164617:c.G1354A:p.V452M | 0.01034- Latino/Admixed American/ SIFT: deleterious (0.02), PolyPhen2: B (0.149), REVEL: 0.158 (B) | Hem | XLR | B/ B-LB/ B [Sprissler et al., 2020] |
|  |  |  |  |  |  |  |  |  |  |  | RXFP2:NM_130806:c.C562A:p.Q188K | 0.0003273- Other/ SIFT: tolerated (0.66), PolyPhen2: B (0.034), REVEL: 0.144 (B) | Het | AR | VUS/ NA/ NA |
| **94 #**  12.5 Y  M | NA | NA | NA | NA | NA | Buried micropenis (2cm), epispadias, urethral orifice, developed scrotum, BC, NPRG | No Müllerian ducts | R: inguinal, L: inguinal | R: hypotrophic testis/ NA, L: testis/ NA | Macrocephaly, hyperopias, fleshy lip, broad nasal bridge, anteverted nares, low-set ears, puffy fingers, small hands, campomelic dysplasia | ROR2:NM_004560:c.T332A:p.I111N | Novel/ SIFT: deleterious (0.02), PolyPhen2: probably damaging (0.972), REVEL: 0.659 (LDC) | Hom | AR | P/ NA/ NA |
|  |  |  |  |  |  |  |  |  |  |  | MAP3K1:NM_005921:c.C1108T:p.P370S | 0.0001292- African/African-American/ SIFT: tolerated (0.09), PolyPhen2: B (0.021), REVEL: 0.044 (B) | Het | AD | VUS/ NA/ NA |
|  |  |  |  |  |  |  |  |  |  |  | GPRC6A:NM_148963:c.A1528C:p.N510H | 0.003367- African/African-American/ SIFT: tolerated (0.52), PolyPhen2: B (0.003), REVEL: 0.215 (B) | Het |  | VUS/ NA/ NA |
|  |  |  |  |  |  |  |  |  |  |  | SLC29A3:NM_001174098:c.C707T:p.T236M | 0.0009799- South Asian/ SIFT: deleterious (0.02), PolyPhen2: B (0.393), REVEL: 0.264 (B) | Het | AR | VUS/ VUS/ NA |
|  |  |  |  |  |  |  |  |  |  |  | AKR1C4:NM_001818:c.G227C:p.R76T | Novel/ SIFT: deleterious (0), PolyPhen2: possibly damaging (0.907), REVEL: 0.682 (LDC) | Het | AR | VUS/ NA/ VUS [Penning, 2015] |
| **95 #**  12.5 Y  M | 3.46 [0.4-4.6] (13Y) | 2.92 [0.1-7.8] (13Y) | 2.23 [0.2-3] (13Y) | NA | NA | Phallus (4.5cm), epispadias, developed scrotum, UC, NPG | No Müllerian ducts | R: inguinal, L: scrotum | R: testis/ NA, L: testis/ NA | Height and weight growth delay, brachydactyly, cardiopathy | SOS1:NM_005633:c.C73T:p.P25S | 0.002280- African/African-American/ SIFT: tolerated (0.68), PolyPhen2: B (0), REVEL score: 0.210 (B) | Het | AD | LP/ B/ B [Kanavy et al., 2019] |
| **97 #**  4 Y  M | NA | NA | <0.025 [0.03-0.38] (4Y) | 0.21 [0.03-0.38] (4Y) | 10.5 [16.8-193] (4Y) | Micropenis (2.5cm), developed scrotum, BC, NPG | No Müllerian ducts | R: inguinal, L: no residual gonad | R: proably hypotrophic testis/ NA, L: no residual gonad | Psychomotor retardation, cerebralpalsy | CHD7:NM_017780:c.T3301C:p.C1101R | Novel/ SIFT: deleterious (0), PolyPhen2: probably damaging (0.968), REVEL: 0.934 (LDC) | Het | AD *De novo or* maternal | LP/ NA/ P [Bergman et al., 2012] |
| **98 #**  2 Mo  F | 0.65 [<3] (3Mo) | 0.80 [<1] (3Mo) | 0.76 [0.03-0.38] (3Mo) | 6.77 [0.03-0.38] (5Mo) | 194 [42-203] (3Mo) | Micropenis (1cm), perineal hyposadias, uretralorifice, hypoplastic bifid depigmented scrotum, NPLG | No Müllerian ducts | R: inguinal, L: inguinal | R: testis/ NA, L: testis/ NA | Cortical and subcortical atrophy | AR:NM_000044:c.G1768T:p.G590W | Novel/ SIFT: deleterious (0), PolyPhen2: probably damaging (1), REVEL: 0.921 (LDC) | Hem | XLR | P/ NA/ NA |
|  |  |  |  |  |  |  |  |  |  |  | FGFR1:NM_001174067:c.G659A:p.R220H | 0.00008827- European (non-Finnish)/ SIFT: tolerated (0.29), PolyPhen2: probably damaging (0.93), REVEL: 0.236 (B) | Het | AD Paternal | LP/ NA/ NA |
| **99**  21 Mo  M | NA | NA | <0.025 [0.03-0.38] (2Y) | 4.44 [0.03-0.38] (2Y) | 71.7 [21-210] (2Y) | Micropenis (3cm), penoscrotal hypospadias, developed scrotum, BC, NPRG | No Müllerian ducts | R: inguinal, L: inguinal | R: testis/ NA, L: testis/ NA | Congenital hip dysplasia | NIPBL:NM_015384:c.6954+3A>G | 0.0001693- Latino/Admixed American/ LOF | Het | AD | VUS/ NA/ P [Krawczynska et al., 2019] |
| **100 #**  29 Mo  M | NA | NA | <0.025 [0.03-0.32] (3Y) | NA | 111.14 [3.8-159] (3Y) | Curved micropenis (2cm), penoscrotal hypospadias, urogenital sinus partially closed, developed scrotum, BC, PG | No Müllerian ducts | R: inguinal, L: inguinal | R: testis/ NA, L: testis/ NA | Height and weight growth delays, chubby facies, pectus excavatum, right uretero-hydronephrosis | OFD1:NM_003611:c.A2044C:p.I682L | 0.006875- African/African-American/ SIFT: tolerated (0.43), PolyPhen2: B (0.013), REVEL: 0.156 (B) | Hem | XLR | P/ B/ NA |
|  |  |  |  |  |  |  |  |  |  |  | HOXA13:NM_000522:c.G1024A:p.A342T | Novel/ SIFT: tolerated (0.11), PolyPhen2: B (0.251), REVEL: 0.526 (LDC) | Het | AD Maternal | P/ NA/ NA |
|  |  |  |  |  |  |  |  |  |  |  | KAT6B:NM_012330:c.C1754T:p.A585V | 0.00009649- Ashkenazi Jewish/ SIFT: tolerated low confidence (0.22), PolyPhen2: B (0.001), REVEL: 0.028 (B) | Het | AD Maternal | VUS/ NA/ NA |
|  |  |  |  |  |  |  |  |  |  |  | FANCB:NM_001018113:c.A388G:p.M130V | 0.0002111- African/African-American/ SIFT: deleterious (0), PolyPhen2: B (0), REVEL: 0.036 (B) | Hem | XLR | P/ LB-B/ NA |
|  |  |  |  |  |  |  |  |  |  |  | GHRHR:NM_000823:c.269-4C>T | 0.0003509- East Asian/ LOF | Het | AR Paternal | VUS/ NA/ NA |
|  |  |  |  |  |  |  |  |  |  |  | SRA1:NM_001035235:c.T536C:p.I179T | 0.009745- Ashkenazi Jewish/ SIFT: deleterious (0.02), PolyPhen2: probably damaging (0.989), REVEL: 0.657(LDC) | Het | AD Maternal | VUS/ NA/ LP [Neocleous et al., 2020] |
| **101 #**  3 Y  M | NA | NA | <0.025 [0.03-0.38] (3.5Y) | 5.93 [0.03-0.38] (3.5Y) | 247 [16.8-193] (3Y) | Phallus (4cm), urogenital sinus closed, hypoplastic scrotum, PG | No Müllerianducts | R: inguinal, L: inguinal | R: testis/ NA, L: testis/ NA | Height, weight, growth delay, myopia, strabismus, inguinal hernia | MPDZ:NM_001261407:c.G2344A:p.G782R | 0.02974- Ashkenazi Jewish/ SIFT: deleterious (0), PolyPhen2: probably damaging (1), REVEL: 0.533 (LDC) | Hom | AR | B/ LB- VUS/ VUS [Ali et al., 2011] |
|  |  |  |  |  |  |  |  |  |  |  | SRA1:NM_001035235:c.C218T:p.P73L | 0.01544- African/African-American/ SIFT: deleterious (0.04), PolyPhen2: possibly damaging (0.502), REVEL: 0.196 (B) | Het | AR | VUS/ NA/ NA |
|  |  |  |  |  |  |  |  |  |  |  | CACNA1F:NM_005183:c.C4360G:p.L1454V | Novel/ SIFT: deleterious (0), PolyPhen2: probably damaging (0.976), REVEL: 0.788 (LDC) | Hem | XLR | P/ NA/ NA |
| **102 #**  7.5 Y  M | NA | NA | 0.22 [0.05-7] (8Y) | NA | 115.8 [11.2-141] (8Y) | Curved micropenis (1cm), anterior hypospadias, developed scrotum | No Müllerian ducts | R: scrotum, L: scrotum | R: testis/ NA, L: testis/ NA | Obesity, retinitis pigmentosa, retrognathism, polydactyly, renal anomalies, intellectual disability | BBS7:NM_176824:c.963delC:p.P321fs | Novel/ LOF | Hom | AR | P/ NA/ NA |
|  |  |  |  |  |  |  |  |  |  |  | GPRC6A:NM_148963:c.2323dupT:p.Y775fs | 0.2768- African/African-American/ LOF | Het |  | B/B/VUS [Dawson et al., 2020] |
|  |  |  |  |  |  |  |  |  |  |  | PROKR2:NM_144773:c.C253T:p.R85C | 0.002218- Ashkenazi Jewish/ SIFT: deleterious (0), PolyPhen2: probably damaging (1), REVEL: 0.422 (B) | Het | AD | LP/ LP/ P[McCormack et al., 2017] |
| **103**  6.5 Y  M | NA | NA | NA | NA | NA | Micropenis (2cm), urogenital sinus closed, urethral orifice, developed scrotum, NPG | No Müllerian ducts | R: inguinal, L: inguinal | R: testis/ NA, L: testis/ NA | Height and weight growth delays, autism, epilepsy | NR0B1:NM_000475:c.G979A:p.E327K | 0.0001463- Latino/Admixed American/ SIFT: tolerated (0.05), PolyPhen2: probably damaging (0.979), REVEL: 0.361 (B) | Hem | XL | VUS/ NA/ NA |
|  |  |  |  |  |  |  |  |  |  |  | SRA1:NM_001035235:c.C59T:p.P20L | 0.0002111- Other/ SIFT: deleterious (0), PolyPhen2: probably damaging (0.997), REVEL: 0.262 (B) | Het | AR | VUS/ NA/ VUS[Kotan et al., 2016] |
|  |  |  |  |  |  |  |  |  |  |  | SOX8:NM_014587:c.G1144A:p.D382N | 0.001036- European (non-Finnish)/ SIFT: deleterious (0.01), PolyPhen2: possibly damaging (0.84), REVEL: 0.722 (LDC) | Het | AD | VUS/ NA/ P[Portnoi et al., 2018] |
| **104 #**  27 Mo  M | NA | NA | NA | 1.55 [<0.2] (28Mo) | 38.0 [16.8-193] (28Mo) | Buried micropenis (<0.5cm), poorly developed and fused labioscrotal folds, BC, NPG | No Müllerian ducts | R: abdominal cavity, L: abdominal cavity | R: testis/ NA, L: testis/ NA | Height, growth delay, facial dysmorphia, hirsutism, quadriparesis, hepatomegaly, cortical atrophy, thrombocytosis, west syndrome | NGLY1:NM_018297:c.G731A:p.W244X | Novel/ LOF | Hom | AR | P/ NA/ NA |
|  |  |  |  |  |  |  |  |  |  |  | DHX37:NM_032656:c.G1460A:p.R487H | 0.0001638- Other/ SIFT: deleterious (0.04), PolyPhen2: B (0.014), REVEL: 0.159 (B) | Het | AD Paternal | VUS/ LP/ LP[Karaca et al., 2015] |
|  |  |  |  |  |  |  |  |  |  |  | AMH:NM_000479:c.C1556T:p.A519V | 0.002603- European (non-Finnish)/ SIFT: tolerated (1), PolyPhen2: B (0.219), REVEL: 0.066 (B) | Het | AR | LB/ LB/ B[Gorsic et al., 2017] |
|  |  |  |  |  |  |  |  |  |  |  | MYRF:NM_001127392:c.C2227T:p.P743S | 0.0004008- Latino/Admixed American/ SIFT: tolerated (0.17), PolyPhen2: B (0.082), REVEL: 0.047 (B) | Het | AD *De novo* or maternal | VUS/ NA/ NA |
| **105 #**  8 Y  M | NA | NA | 0.07 [0.025-0.38] (8Y) | NA | 43.6 [11.2-141] (8Y) | Penis (6cm), , urethral orifice, developed scrotum, BC, NPG | No Müllerian ducts | R: inguinal, L: inguinal | R: testis/ NA, L: testis/ NA | Strabismus, psychomotor delay, writing difficulties | FOXP1:NM_001244813:c.C1203G:p.Y401X | Novel/ LOF | Het | AD *De novo* | P/ NA/ NA |
| **106 #**  2 D  M | 0.554 [<1.5] (23D) | 5.44 [<1.5] (23D) | 2.93 [<0.20] (23D) | NA | 123.82 [16.8-138] (23D) | Curved micropenis (2.5cm), urogenital sinus closed, urethral orifice, BC, PG | No Müllerian ducts | R: inguinal, L: inguinal | R: testis/ NA, L: testis/ NA | Right subangulomaxillary subcutaneous fat, inguinal hernia | AR:NM_000044:c.T1833A:p.N611K | Novel/ SIFT: deleterious (0), PolyPhen2: probably damaging (1), REVEL: 0.647 (LDC) | Hem | AR | P/ NA/ NA |
|  |  |  |  |  |  |  |  |  |  |  | GPRC6A:NM_148963:c.G670T:p.A224S | 0.0001147- African/ African-American/ SIFT: deleterious (0), PolyPhen2: probably damaging (0.936), REVEL: 0.506 (LDC) | Het |  | VUS/ NA/ NA |
|  |  |  |  |  |  |  |  |  |  |  | ROR2:NM_004560:c.G1583A:p.R528Q | 0.003128- African/ African- American/ SIFT: deleterious (0.04), PolyPhen2: possibly damaging (0.899), REVEL: 0.495 (B) | Het | AR | VUS/ VUS/ P[Aglan et al., 2015] |
|  |  |  |  |  |  |  |  |  |  |  | FLNA:NM_001110556:c.C7450T:p.R2484C | Novel/ SIFT: deleterious (0.01), PolyPhen2: possibly damaging (0.834), REVEL: 0.592 (LDC) | Hem | XLD | VUS/ NA/ VUS[DiFrancesco et al., 2019] |
| **107 #**  2D  M | 2.14 [0.30-11.0] (24Mo) | 0.80 [0.10-0.50] (24Mo) | 0.15 [1.56-8.77] (5Y) | NA | 0.08 [0.38-159.8] (8Y) | Micropenis (1cm), posterior hypospadias, urethral orifice, hypoplastic depigmented bifid labioscrotal folds, NPG | No Müllerian ducts | R: inguinal, L: inguinal | R: testis/ NA, L: hypotrophic testis/ NA | Height and weight growth delays, PSIS | GLI3:NM_000168:c.T3935G:p.M1312R | 0.001101- Latino/Admixed American/ SIFT: tolerated low confidence (0.06), PolyPhen2: B (0.0), REVEL: 0.068 (B) | Het | AD | P/ B-LB/ NA |
|  |  |  |  |  |  |  |  |  |  |  | POR:NM_000941:c.C683T:p.P228L | Novel/ SIFT: deleterious (0), PolyPhen2: probably damaging (0.966), REVEL: 0.549 (LDC) | Het | AR | VUS/ NA/ VUS [Kolesinska et al., 2018] |
| **108**  5.5 Y  M | NA | NA | <0.025 [0.03-0.38] (5.5Y) | NA | 62 [12.6-167] (5.5Y) | Micropenis, hypospadias, urethral orifice, developed scrotum, PG | No Müllerian ducts | R: scrotum, L: scrotum | R: oscillating testis/ NA, L: oscillating testis/ NA | Tetralogy of Fallot | HHAT:NM_001170587:c.A623G:p.Y208C | Novel/ SIFT: deleterious (0), PolyPhen2: probably damaging (0.902), REVEL: 0.856 (LDC) | Het | AR Maternal | VUS/ NA/ NA |
| **109 #**  2 D  M | 1.7 [0.95-11.95] (3.5Y) | 0.09 [1.14-8.75] (3.5Y) | <0.08 [1.5-5.77] (3.5Y) | 4.16 [<0.3] (11Y) | 40.4 [51.3-88.3] (3.5Y) | Penis (4cm), posterior hypospadias, urogenital sinus partially closed, urethral orifice, lower distal end of the urethra marked by the presence of a second orifice, bifid depigmented labioscrotal folds, BC, NPRG | No Müllerian ducts | R: inguinal, L: inguinal | R: testis (12.7x9.5x10mm)/ NA, L: testis (11x7x5mm)/ NA | Height and weight growth delays, simple splenic cystic formation, pachyvaginalitis | PTCH1:NM_000264:c.C37G:p.R13G | 0.001016- East Asian/ SIFT: tolerated low confidence (0.37), PolyPhen2: B (0), REVEL: 0.259 (B) | Het | AD | VUS/ B- LB- VUS/ NA |
|  |  |  |  |  |  |  |  |  |  |  | PTCH1:NM_000264:c.G2635A:p.D879N | 0.0001006- European (non-Finnish)/ SIFT: deleterious (0), PolyPhen2: possibly damaging (0.687), REVEL: 0.561 (LDC) | Het | AD | VUS/ VUS/ VUS[Ishida et al., 2018] |
|  |  |  |  |  |  |  |  |  |  |  | PTCH1:NM_000264:c.C4151T:p.P1384L | 0.0001309- South Asian/ SIFT: tolerated low confidence (0.15), PolyPhen2: B (0.308), REVEL: 0.222 (B) | Het | AD | VUS/ LB- VUS/ NA |
|  |  |  |  |  |  |  |  |  |  |  | RXFP2:NM_130806:c.C958A:p.L320I | Novel/ SIFT: tolerated (0.2), PolyPhen2: possibly damaging (0.531), REVEL: 0.141 (B) | Hom | AR | P/ NA/ NA |
|  |  |  |  |  |  |  |  |  |  |  | LHX3:NM_014564:c.G221A:p.C74Y | Novel/ SIFT: deleterious (0), PolyPhen2: probably damaging (1), REVEL: 0.916 (LDC) | Het | AR | VUS/ NA/ NA |
|  |  |  |  |  |  |  |  |  |  |  | HSD17B6:NM_003725:c.G285T:p.Q95H | 0.0004687- Latino/Admixed American/ SIFT: deleterious (0.01), PolyPhen2: B (0.171), REVEL: 0.337 (B) | Het | AR | VUS/ NA/ NA |
| **110**  5 Y  M | NA | NA | <0.025 [0.03-0.38] (5Y) | NA | 38.5 [126-167] (5Y) | Penis (4cm), urogenital sinus closed, hypoplastic scrotum, NPRG | No Müllerian ducts | R: inguinal, L: inguinal | R: testis/ NA, L: testis/ NA | Height, weight growth delay, chubby facies, empty saddle turcica with anterior pituitary atrophy | KAT6B:NM_001256468:c.G1546A:p.A516T | 0.0002803- African/African-American/ SIFT: tolerated (0.18), PolyPhen2: possibly damaging (0.902), REVEL score: 0.330 (B) | Het | AD | VUS/ NA/ NA |
| **111**  9 Y  M | NA | NA | 0.0577 [0.86-3.06] (9Y) | NA | 68.83 [38.25-332.4] (9Y) | Micropenis, urethral orifice, penoscrotal hypospadias, developed scrotum | No Müllerian ducts | R: scrotum, L: scrotum | R: testis/ NA, L: testis/ NA | Myopia, hypertrophic right kidney, mute left kidney | ZNRF3:NM_001206998:c.G1507A:p.G503S | 0.0005872- Other/ SIFT: tolerated (0.13), PolyPhen2: B (0.097), REVEL score: 0.144 (B) | Het |  | VUS/ VUS/ NA |
| **112 #**  30 Mo  M | NA | NA | 0.12 [0.0-1.0] (32Mo) | 6.46 [1.43-11.6] (34Mo) | 23 [16.8-193] (32Mo) | Curved micropenis (2.5cm), perineal hypospadias, urogenital sinus opened, urethral orifice, hypoplastic scrotum, NPRG | No Müllerian ducts | R: inguinal, L: inguinal | R: testis/ NA, L: testis/ NA | Height; weight growth delay, hand deformity, vesicoureteric reflux grade III, anorectal malformation | CHD7:NM_017780:c.G1030A:p.V344I | 0.0001922- East Asian/ SIFT: tolerated low confidence (0.57), PolyPhen2: possibly damaging (0.796), REVEL score: 0.204 (B) | Het | AD | LP/ LB/ VUS [Ittisoponpisan and David et al., 2018] |
|  |  |  |  |  |  |  |  |  |  |  | SLC29A3:NM_001174098:c.C707T:p.T236M | 0.0009799- South Asian/ SIFT: deleterious (0.02), PolyPhen2: B (0.393), REVEL score: 0.264 (B) | Het | AR | VUS/ VUS/ NA |
| **113 #**  2 D  M | 25.94 [0.95-11.95] (5.5Mo) | 2.08 [0.57-12.07] (5.5Mo) | 0.6 [3.0-12.0] (5D) | NA | <0.08 [3.8-159.8] (5.5Mo) | Genital bud (1cm), hypospadias, bifid labioscrotal folds | Uterus present, vaginal cavity present | No residual gonad | No residual gonad | Congenital rib dislocation vertebral dysplasia | GLI3:NM_000168:c.T3935G:p.M1312R | 0.001101- Latino/Admixed American/ SIFT: tolerated low confidence (0.06), PolyPhen2: B (0.0), REVEL: 0.068 (B) | Het | AD Paternal | LP/ B-LB-VUS/ NA |
|  |  |  |  |  |  |  |  |  |  |  | CTU2:NM_001012759:c.C1439T:p.P480L | 0.0001151- African/ African American/ SIFT: deleterious (0), PolyPhen2: probably damaging (1), REVEL: 0.307 (B) | Het | AR Maternal | VUS/ NA/ NA |
| **114**  2 D  M | 0.05 [1-12] (50D) | 0.09 [1.5-9.3] (50D) | 0.225 [0.5-4.7] (50D) | 0.16 [3.0-12.0] (2.5M) | 76.5 [35-95.1] (50D) | Micropenis (5mm), perineal hypospadias, asymmetric scrotum, UC, NPLG | No Müllerian ducts | R: scrotum, L: inguinal | R: hypotrophic testis/ NA, L: hypotrophic testis/ NA | Facial asymmetry, inguinal hernia in the right | FEZF1:NM_001024613:c.T553C:p.F185L | 0.003758- South Asian/ SIFT: tolerated (0.69), PolyPhen2: B (0), REVEL score: 0.091 (B) | Het | AR Paternal | VUS/ B- LB/ NA |
|  |  |  |  |  |  |  |  |  |  |  | DCAF17:NM_025000:c.1422+3G>A | 0.0001161- Latino/Admixed American/ LOF | Het | AR Maternal | VUS/ NA/ NA |
| **116 #**  16 Mo  M | 74.8 [1-8] (16Mo) | 11.3 [0.6-12] (16Mo) | 0.02 [3.0-12.0] (16Mo) | 0.2 [3.0-12.0] (20Mo) | <0.01 [21-210] (16Mo) | Genital bud (1.5cm), perineal hypospadias, poorly developed and pigmented labioscrotal folds, BC, NPG | No Müllerian ducts | No residual gonad | No residual gonad | Saddle nose, broad rounded forehead | GLI3:NM_000168:c.G2179A:p.G727R | 0.007763- European (Finnish)/ SIFT: deleterious (0.04), PolyPhen2: possibly damaging (0.803), REVEL: 0.467 (B) | Hom | AD | P/ B-LB- LP/ P [Szczepanek-Parulska et al., 2021] |
|  |  |  |  |  |  |  |  |  |  |  | MAMLD1:NM_001177465:c.G1793A:p.R598H | 0.0009965- African/ African-American/ SIFT: deleterious (0), PolyPhen2: probably damaging (0.982), REVEL: 0.183 (B) | Hem | XLR | VUS/ NA/ NA |
| **117 #**  3 Mo  M | 0.49 [1-8] (16Mo) | 0.7 [0.6-12] (16Mo) | 1.21 [3.0-12.0] (3Mo) | 6.54 [3.0-12.0] (21Mo) | 164.4 [39.1-91.1] (3Mo) | Penis (4cm), perineal orifice, bifid depigmented labioscrotal folds, BC, NPG | Uterus, vaginal cavity, female urethra | R: abdominal cavity, L: abdominal cavity | R: testis/ NA, L: testis/ NA | Mental retardation, bilateral inguinal hernia | SLC29A3:NM_018344:c.A1001G:p.N334S | 0.007734- African/African-American/ SIFT: tolerated (0.99), PolyPhen2: B (0.007), REVEL: 0.110 (B) | Hom | AR | P/ B-LB/ P[Noavar et al., 2019] |
|  |  |  |  |  |  |  |  |  |  |  | FANCD2:NM_033084:c.T2255C:p.I752T | Novel/ SIFT: deleterious (0.03), PolyPhen2: possibly damaging (0.791), REVEL: 0.578 (LDC) | Het | AR | VUS/ NA/ NA |
| **118 #**  3 D  M | 7.53 (2Mo) | 13.53 (2Mo) | 1.15 [3.0-12.0] (2Mo) | NA | 70.3 [39-91] (2Mo) | Curved genital bud (1.5cm), posterior orifice, hypoplatic scrotum, BC, NPG | No Müllerian ducts | R: abdominal cavity, L: abdominal cavity | R: infracentimetric testis with total epididymo-testicular nonunion/ NA, L: testis/ NA | Height, growth delay, bilateral congenital aniridia, bilateral nystagmus, cortical atrophy, psychomotor delay, asthma, compressive mediastinal pulmonary adenomegaly, hepatospleno-megaly, supernumerary spleen, myelodys-plastic syndrome, hyperleuko-cytosis and thrombo-cytopenia, immune deficiency, cafe-au-lait spots | MAMLD1:NM_001177465:c.C2573T:p.P858L | Novel/ SIFT: deleterious (0.03), PolyPhen2: possibly damaging (0.625), REVEL: 0.188 (B) | Hem | XLR | VUS/ NA/ NA |
|  |  |  |  |  |  |  |  |  |  |  | NRAS:NM_002524:c.G35C:p.G12A | 0.00004619- European (Finnish)/ SIFT: deleterious (0.02), PolyPhen2: possibly damaging (0.525), REVEL: 0.689 (LDC) | Het | AD *De novo* | P/ P/ P [Sadlecki et al., 2018] |
|  |  |  |  |  |  |  |  |  |  |  | SHH:NM_000193:c.G277A:p.G93R | 0.00003266- South Asian/ SIFT: deleterious (0), PolyPhen2: probably damaging, REVEL: 0.922 (LDC) | Het | AD Maternal | P/ NA/ NA |
|  |  |  |  |  |  |  |  |  |  |  | RXFP2:NM_130806:c.G184A:p.A62T | 0.001524- Other/ SIFT: tolerated (0.43), PolyPhen2: B (0.035), REVEL: 0.281 (B) | Het | AR Maternal | VUS/ NA/ NA |
| **119 #**  2 D  M | NA | NA | 0.007 [3.0-12.0] (20Mo) | 1.92 [3.0-12.0] (21Mo) | 20.7 [51.3-88.3] (19Mo) | Micropenis (2.5cm), penoscrotal hypospadias, BC, NPG | Müllerian ducts present, male urethra | R: no residual gonad, L: abdominal cavity | R: no residual gonad, L: testis/ NA | Splenic cystic formation, left pelvic cyst formation | FSHB:NM_001018080:c.G107A:p.R36H | 0.00004624- European (Finnish)/ SIFT: tolerated (0.17), PolyPhen2: B (0.001), REVEL: 0.215 (B) | Het | AR | VUS/ NA/ NA |
|  |  |  |  |  |  |  |  |  |  |  | RXFP2:NM_130806:c.C1594G:p.R532G | 0.00004619- European (Finnish)/ SIFT: tolerated (0.07), PolyPhen2: B (0.168), REVEL: 0.248 (B) | Het | AR | VUS/ NA/ NA |
|  |  |  |  |  |  |  |  |  |  |  | LHX3:NM_014564:c.C1130T:p.T377M | 0.0002942- Other/ SIFT: deleterious (0), PolyPhen2: probably damaging (0.985), REVEL: 0.475 (B) | Het | AR | VUS/ NA/ NA |
|  |  |  |  |  |  |  |  |  |  |  | GPRC6A:NM_148963:c.A1447C:p.N483H | Novel/ SIFT: deleterious (0.03), PolyPhen2: B (0.332), REVEL: 0.340 (B) | Het |  | VUS/ NA/ NA |
|  |  |  |  |  |  |  |  |  |  |  | MYRF:NM_001127392:c.C2227T:p.P743S | 0.0004008- Latino/Admixed American/ SIFT: tolerated (0.17), PolyPhen2: B (0.082), REVEL: 0.047 (B) | Hom | AD | LP/ NA/ NA |
| **120 #**  4.5 Y  M | 0.56 [1-8] (4.5Y) | 0.29 [0.6-12] (4.5Y) | 0.01 [3.0-12.0] (4.5Y) | 2.19 [3.0-12.0] (4.5Y) | 17.22 [51.3-88.3] (4.5Y) | Genital bud (3cm), perineal hypospadias, BC, NPG | Müllerian ducts present | R: inguinal, L: inguinal | R: hypotrophic testis/ NA, L: hypotrophic testis/ NA | Small head, broad forehead, retrognatism, low-set ears, dental caries, clinodactyly, cerebral hypoplasia, PSIS, chronic diarrhea | GHR:NM_001242399:c.A1601T:p.N534I | Novel/ SIFT: deleterious (0.02), PolyPhen2: possibly damaging (0.832), REVEL: 0.234 (B) | Het | AD Maternal | VUS/ NA/ NA |
|  |  |  |  |  |  |  |  |  |  |  | NIPBL:NM_015384:c.A2461G:p.K821E | Novel/ SIFT: tolerated low confidence (0.09), PolyPhen2:B (0.14), REVEL: 0.356 (B) | Het | AD Maternal | P/ NA/ NA |
| **121 #**  7.5 Mo  M | 4.8 [1-8] (8Mo) | 3.7 [0.6-12] (8Mo) | 1.72 [3.0-12.0] (8Mo) | NA | 40.26 [21-210] (7.5Mo) | Genital bud (2.5cm), perineal hypospadias, developed scrotum, PG | No Müllerian ducts | R: scrotum, L: scrotum | R: testis (8.5mm)/ NA, L : testis (8.5mm)/ NA | Short stature, history of dehydration, severe undernutrition hypertelorism, discreet supratentorial hydrocephalus, cranial dysmorphism, sequelae cortico-subcortical atrophy, ostium secundum | SRA1:NM_001035235:c.T536C:p.I179T | 0.009745- Ashkenazi Jewish/ SIFT: deleterious (0.02), PolyPhen2: probably damaging (0.989), REVEL: 0.657(LDC) | Het | AR | VUS/ NA/ LP[Neocleous et al., 2020] |
|  |  |  |  |  |  |  |  |  |  |  | AMH:NM_000479:c.C553G:p.Q185E | 0.003098- Ashkenazi Jewish/ SIFT: deleterious (0.01), PolyPhen2: probably damaging (0.925), REVEL: 0.492 (B) | Het | AR | VUS/ NA/ VUS |
|  |  |  |  |  |  |  |  |  |  |  | FGFR3:NM_001163213:c.C2339G:p.P780R | Novel/ SIFT: tolerated (0.84), PolyPhen2: B (0.003), REVEL: 0.173 (B) | Het | AD Paternal | LP/ NA/ NA |
| **122 #**  9.5 Y  M | 2.5 (9.5Y) | 1.39 (9.5Y) | 0.16 [1.5-8.77] (9.5Y) | NA | 54.9 [11.2-141] (9.5Y) | Micropenis (3.5cm), penoscrotal hypospadias, urethral orifice, hypoplastic pigmented scrotum, NPG | No Müllerian ducts | No residual gonad | No residual gonad | High growth delay, hydrocephalus, short neck | GPC3:NM_001164617:c.A1709G:p.N570S | Novel/ SIFT: tolerated (0.57), PolyPhen2: B (0), REVEL: 0.038 (B) | Hem | XLR | P/ NA/ NA |
|  |  |  |  |  |  |  |  |  |  |  | PRKAR1A:NM_001278433:c.G221A:p.R74H | 0.001524- Other/ SIFT: tolerated (0.17), PolyPhen2: B (0.031), REVEL: 0.382 (B) | Het | AD | LP/ LB- VUS/ P [Tsay et al., 2017] |

**ACMG** American College of Medical Genetics, **AD** autosomal dominant, **AMH** anti-Müllerian hormone, **AR** autosomal recessive, **B** benign, **BC** bilateral cryptorchidism, **CAIS** complete androgen insensitivity syndrome, **D** day, **DSD** disorders/differences of sex development, **F** female, **FSH** follicle stimulating hormone, **GD** gonadal dysgenesis, **gnomAD** genome aggregation database, **GV** gene variants previously associated with the disease, **HCG** human chorionic gonadotropins, **Hem** hemizygous, **Het** heterozygous, **Hom** homozygous, **L** left, **LB** likely benign, **LDC** likely disease causing, **LH** luteinizing hormone, **LOF** loss-of-function, **LP** likely pathogenic, **LPG** left palpable gonad, **M** male, **MAF** minor allele frequency, **Mo** month, **NA** not available, **NPG** non palpable gonad**s**, **NPLG** non palpable left gonad, **NPRG** non palpable right gonad, **NR** normal range, **P** pathogenic, **PAIS** partial androgen insensitivity syndrome, **PG** palpable gonads, **PoGI** Pattern of disease inheritance usually associated with the gene, **R** right, **Ref** reference, **REVEL** rare exome variant ensemble learner, **SRD5A2** steroid 5 alpha-reductase 2, **UC** unilateral cryptorchism, **VUS** variant of uncertain significance, **Y** year. **^#^**A definitive genetic diagnosis was achieved
